# Comparative analysis of drying kinetics, thermodynamic properties, and mathematical modeling of pomegranate peel (Punica granatum L.) in a hybrid solar dryer and an oven dryer

**DOI:** 10.1038/s41598-025-11016-8

**Published:** 2025-07-19

**Authors:** El-Sayed G. Khater, Adel H. Bahnasawy, Abdallah Elshawadfy Elwakeel, Aml Abubakr Tantawy, Ahmed Elbeltagi, Ali Salem, Samy A. Marey, Abdelaziz M. Okasha, Khaled A. Metwally

**Affiliations:** 1 Agricultural and Biosystems Engineering Department, Faculty of Agriculture, Benha University, P.O. Box 13736, Moshtohor, Toukh, Kalubia Egypt; 2 Agricultural Engineering Department, Faculty of Agriculture and Natural resources, Aswan University, Aswan, Egypt; 3 Food Science Department, Faculty of Agriculture, Beni-Suef University, Beni-Suef, 65211 Egypt; 4 Agricultural Engineering Department, Faculty of Agriculture, Mansoura University, Mansoura, 35516 Egypt; 5 Civil Engineering Department, Faculty of Engineering, Minia University, Minia, 61111 Egypt; 6 Structural Diagnostics and Analysis Research Group, Faculty of Engineering and Information Technology, University of Pécs, Pécs, 7622 Hungary; 7 Prince Sultan Bin Abdulaziz International Prize for Water Chair, Prince Sultan Institute for Environmental, Water and Desert Research, King Saud University, Riyadh, 11451 Saudi Arabia; 8 Department of Agricultural Engineering, Faculty of Agriculture, Kafrelsheikh University, Kafr El-Sheikh, 33516 Egypt; 9 Soil and Water Sciences Department, Faculty of Technology and Development, Zagazig University, Zagazig, 44519 Egypt

**Keywords:** Fruit drying, Food by-product, Renewable energy, Thin layer modeling, Effective moisture diffusivity, Plant sciences, Environmental sciences, Energy science and technology

## Abstract

Drying pomegranate peels, a by-product of juice extraction, offers an effective means to preserve bioactive compounds while reducing waste. This study investigates the influence of drying temperature and layer thickness on the drying kinetics, thermodynamic properties, and modeling of pomegranate peels using a hybrid solar dryer (HSD) and a conventional oven dryer (OD). Fresh peels with an average initial MC of 325.53% (dry basis) were dried under three temperatures (50, 60, and 70 °C) and three-layer thicknesses (1, 2, and 3 cm). The HSD included a temperature and humidity control unit and an auxiliary electric heater to ensure stable conditions. Weight loss during drying was recorded at regular intervals to track moisture content changes until equilibrium MC (EMC) was reached. Experimental data were used to calculate effective moisture diffusivity (EMD) using Fick’s second law and activation energy through Arrhenius-type relationships. Thermodynamic parameters—Gibbs free energy, enthalpy, and entropy—were also determined. Additionally, twelve thin-layer drying models were fitted to the data using nonlinear regression, with performance evaluated using R², RMSE, and χ². Results showed that final MCs ranged from 2.15 to 2.80% (OD) and 2.92–3.01% (HSD). EMD increased with temperature and thickness, reaching up to 12.17 × 10⁻⁹ m²/s (OD) and 11.66 × 10⁻⁹ m²/s (HSD) at 70 °C for 3 cm layers. Activation energy varied with thickness, ranging from 25.76 to 43.55 kJ/mol (OD) and 25.82 to 41.09 kJ/mol (HSD). Among all models, the Modified Midilli II model best described the drying behavior. Thermodynamic analysis indicated that Gibbs free energy increased with temperature, while enthalpy and entropy decreased, reflecting improved energy efficiency at higher drying temperatures. The results demonstrate that optimized drying conditions can enhance the preservation and quality of pomegranate peels, promoting their use as functional ingredients in the agri-food industry.

**Supplementary Information:**

The online version contains supplementary material available at 10.1038/s41598-025-11016-8.

## Introduction

The pomegranate (Punica granatum L.) originates from Iran and is widely cultivated in countries such as Tunisia, Turkey, Spain, Egypt, Morocco, the United States, China, India, and across Near and East Asia^1–4^. The pomegranate market, valued at USD 248.4 million in 2023, is projected to rise from USD 261.57 million in 2024 to USD 356.55 million by 2032, reflecting a compound annual growth rate (CAGR) of 5.3% between 2025 and 2032^5^. Egypt, Spain and Israel are the most prominent exporting countries, but the Egyptian crop is of great importance in international contracts due to its high quality and the organization of the export process. The high demand for Egyptian pomegranates has contributed to raising its prices to record levels, especially in the markets of Europe, Russia and Arab countries, most notably Iraq^6^. Pomegranate is esteemed for its nutritional properties and is frequently utilized in juice and wine manufacturing. The processing of pomegranates produces significant byproducts, mainly including peels and seeds, with the peel representing 50% of the fresh fruit weight^7^. Pomegranate peel is notably abundant in phenolic compounds, such as punicalagin, ellagic acid, gallic acid, and rutin, with concentrations surpassing those present in the fruit pulp^8,9^. These chemicals have a range of advantageous qualities, including anti-cancer^10^anti-inflammatory^11^antibacterial^12^antioxidant, and hypoglycemic effects^13^. As a result, pomegranate peels have gained extensive utilization in the pharmaceutical, cosmetic, and food sectors^14^.

Pomegranate peels can be dried using various methods, each affecting the quality, efficiency, and nutritional value of the final product. Common techniques include sun drying, oven drying, and solar drying. Solar drying of pomegranate peels is an eco-friendly and cost-effective method to preserve and utilize this often-discarded agricultural byproduct^15–17^. By harnessing solar energy, the process reduces MC, preventing spoilage and extending shelf life while retaining valuable bioactive compounds like polyphenols and antioxidants^18–20^. Solar drying is energy-efficient, sustainable, and accessible, making it ideal for small-scale farmers and processors^21–24^. Dried pomegranate peels can be used in food, pharmaceuticals, cosmetics, and agriculture, offering health benefits and economic opportunities. This approach not only reduces waste but also aids environmental protection by decreasing dependence on non-renewable energy sources and fostering a circular economy^25–28^. The development of drying systems, adherence to quality standards, and energy efficiency require assessing the overall quality of dried products and predicting their drying behavior under varying conditions^29–31^. Mathematical modeling plays a pivotal role in drying technology, serving as a foundation for designing and operating dryers under optimal conditions. Since drying affects the physicochemical and quality characteristics of final products, process control strategies often involve simulating drying kinetics^32–34^. Additionally, studying drying kinetics enhances the understanding and quantification of the thermal and physical factors governing the drying process^35–39^. Factors such as air temperature, humidity, product dimensions, and drying time have a significant impact on drying kinetics^26,40,41^. Each aspect must be examined during the drying process, as they may exert differing affects. This problem renders human operation of the dryer system nearly futile. Therefore, developing a model that incorporates multiple variables is crucial for researchers. Numerous drying models—such as Page, Midilli, and logistic models—have been developed to simulate food drying kinetics^42–45^. Understanding material transport in thin-layer drying is essential for effective process simulation and control. Studies show that relying only on experimental methods, without mathematical modeling, can lower dryer efficiency, raise costs, and reduce product quality^46–52^. Thus, robust models are vital for process design, optimization, and control of agricultural product drying^53–55^.

A number of prior academics and scholars have investigated the drying process of pomegranate peels, including Wanderley et al.^56^ investigated the effect of hot air oven drying at 50, 60, and 70 °C on pomegranate peels and seeds. They modeled the drying kinetics using twelve mathematical models, finding the Diffusion Approximation, Verma, and modified Henderson and Pabis models best fit the data. The study determined effective diffusivities and thermodynamic properties, revealing that higher temperatures reduced drying time and that peel flours retained high fiber and mineral content, while seed flours were richer in lipids and proteins Cecchi et al.^57^ reviewed the influence of various industrial drying methods—including sun, microwave, vacuum, and oven drying—on the phenolic and polysaccharide content of pomegranate peel. They highlighted that both the drying technique and extraction protocol (e.g., solvent, supercritical fluid, ultrasound-assisted) significantly affect the recovery of bioactive compounds. For instance, oven drying at 60 °C yielded higher punicalin levels compared to 40 °C, and pressurized liquid extraction achieved the highest total phenolic content, though at higher cost. The review emphasized the need for cost-effective methods that preserve compound. Mphahlele et al.^58^ evaluated the impact of freeze and oven drying (at 40, 50, and 60 °C) on the bioactive, antioxidant, antibacterial, and antityrosinase activities of pomegranate peels. Using colorimetric and chromatographic analyses, they found freeze-dried peels retained the highest total phenolic, tannin, and flavonoid concentrations, while oven drying at 60 °C produced high punicalin but poorer coloration. Both freeze and oven-dried extracts showed significant antibacterial and antioxidant activities, with freeze drying best preserving bioactive. Mphahlele et al.^59^ focused on the drying kinetics of pomegranate peel at 40, 50, and 60 °C with constant air velocity. They fitted ten thin-layer drying models to the data, identifying the Midilli et al. model as the best fit. The study found that drying time decreased with increasing temperature, and EMD and activation energy were quantified, supporting the optimization of drying processes. Galaz et al.^60^ examined double drum drying at 100, 110, and 120 °C, modeling drying kinetics and analyzing polyphenolic content. Drum drying was rapid, achieving low MC in short times without significant loss of total polyphenols or antioxidant activity. Hydrolysable tannins were preserved at lower drum temperatures, suggesting drum drying as an efficient method for high-quality peel processing. And Marchi et al.^61^ compared freeze-drying and oven drying at 60 °C for their effects on antioxidant and antibacterial properties of pomegranate peel extracts. Their findings indicated that freeze-drying better preserved bioactive compounds, resulting in higher antioxidant and antibacterial activities.

To the best of our knowledge, the existing literature lacks comprehensive studies investigating the simultaneous effects of solar drying, drying temperature, and thin-layer thickness on the drying kinetics, thermodynamic properties, and mathematical modeling of pomegranate peels. While individual aspects of drying processes have been explored for various agricultural products, a detailed and integrated analysis focusing specifically on pomegranate peels remains unaddressed. This gap is particularly significant given the growing interest in valorizing fruit by-products for functional and sustainable applications. Therefore, the present study was designed to fill this research gap by employing a hybrid solar dryer (HSD) equipped with advanced control systems for temperature, humidity, and an auxiliary electric heater. This setup enables precise manipulation of drying conditions, ensuring consistent and replicable results. The study examined the drying behavior of pomegranate peels under varying temperatures and thin-layer thicknesses to assess their impact on drying efficiency. Additionally, a comparative analysis was conducted using a conventional oven dryer (OD) to evaluate the performance and effectiveness of the HSD under equivalent conditions. The outcomes of this study are expected to contribute valuable insights into optimizing solar drying processes for pomegranate peels and potentially extend to other similar agro-industrial residues.

This study aligns with multiple Millennium Development Goals (MDGs) by promoting sustainability, enhancing food security, reducing agricultural waste, and adding value to agro-industrial by-products. Specifically, it contributes to MDG 1 (Eradicate Extreme Poverty and Hunger) by converting underutilized agricultural residues, such as pomegranate peels, into value-added products with potential applications in food, pharmaceuticals, and cosmetics—thereby generating income opportunities for small-scale farmers and agro-processors. The implementation of improved drying techniques helps minimize post-harvest losses, preserve food quality, and extend shelf life, directly supporting food availability and nutritional security. In relation to MDG 7 (Ensure Environmental Sustainability), the use of a hybrid solar dryer promotes the adoption of clean, renewable energy sources, reducing reliance on fossil fuels and lowering the carbon footprint of food processing. Moreover, the valorization of agro-waste aligns with sustainable resource management practices. The research also advances MDG 8 (Develop a Global Partnership for Development) by facilitating the transfer of appropriate, cost-effective technologies to developing regions, fostering international collaboration, knowledge exchange, and local capacity building in sustainable agri-food systems. Although indirectly, it also supports MDG 3 (Promote Gender Equality and Empower Women), as women in many developing countries are heavily engaged in food processing. Access to efficient and affordable drying technologies can reduce labor burdens, enhance income potential, and improve working conditions. Collectively, this work contributes to the broader MDG agenda by supporting sustainable agriculture, waste minimization, food preservation, and clean energy adoption—key pillars for improving livelihoods and advancing environmental and socio-economic resilience.

## Materials and methods

### Experimental procedures

The fresh pomegranate fruit (*Origanum majorana L*.) was procured from local markets in Benha, Egypt. The peels were subsequently cleaned to eliminate dirt and impurities, after which they were chopped and extracted from the pomegranates. The average initial MC of pomegranate peels is 325.53% (on dry basis).

The experimental study employed a split-plot design with three replications to investigate the effects of drying method, drying temperature, and sample layer thickness on the drying performance of pomegranate peels (*Origanum majorana L.*). The factors and their respective levels are presented in Table 1.

**Table 1:** Input variables and levels used in the experimental design.

Factor	Type	Levels
Drying Method	Main Plot Factor	Hybrid Solar Dryer (HSD), Oven Dryer (OD)
Drying Temperature (°C)	Sub Plot Factor	50, 60, 70
Sample Thickness (cm)	Sub Plot Factor	1, 2, 3

**Drying Method**:

- *Hybrid Solar Dryer (HSD)*: A flat-plate solar dryer equipped with an auxiliary electric heater.
- *Oven Dryer (OD)*: A conventional hot-air oven dryer used as a control.

#### Drying temperature

Three target drying temperatures were tested—50, 60, and 70 °C—regulated during experiments.

#### Sample thickness

Pomegranate peels were spread in three uniform thicknesses (1 cm, 2 cm, and 3 cm) on the drying trays.

**Fig. 1:**
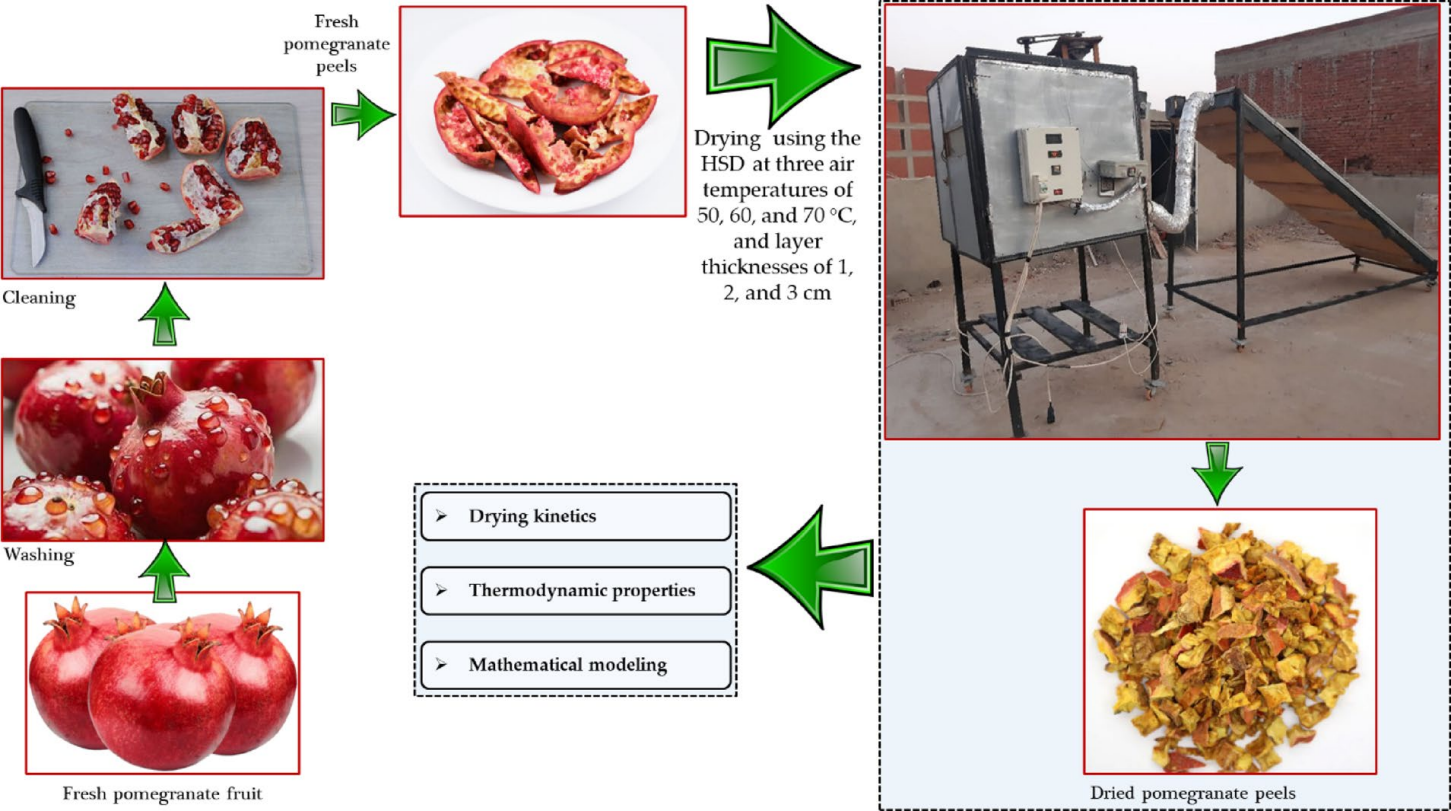
Step-by-step drying process flow diagram using the HSD.

All experiments on the desiccation of fresh pomegranate peels were conducted at the Faculty of Agriculture, Moshtohor, Benha University, Egypt, from May to August 2024. In the outdoor experiments, the ambient air temperature varied from 21.6 to 34.1 °C, relative humidity fluctuated between 42.6 and 77.3%, and solar radiation measured between 321.9 and 954.1 kJ m^− 2^ day^− 1^. Figure 1 shows the step-by-step drying process flow diagram using the HSD.

### Description of the HSD

**Fig. 2:**
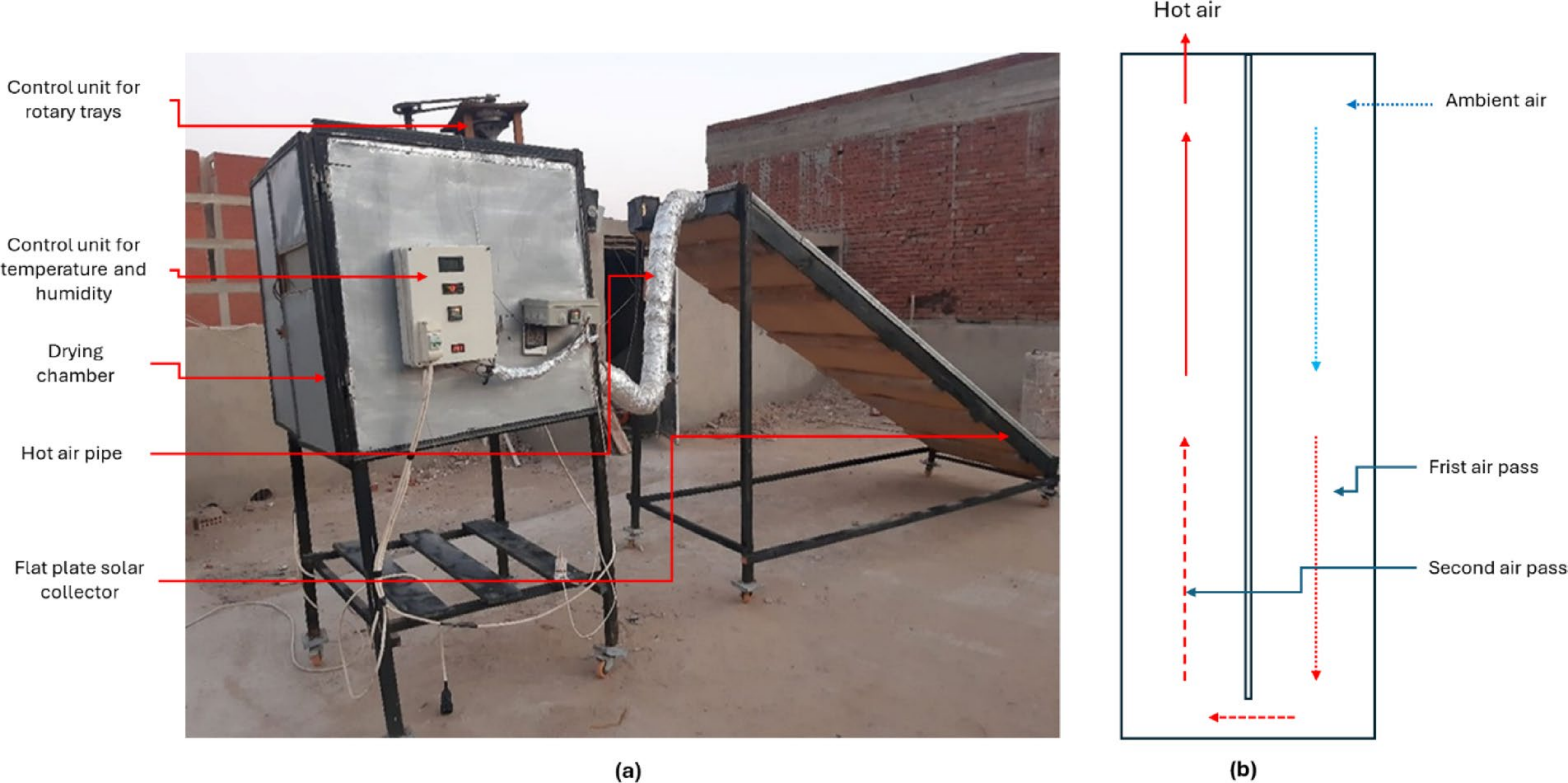
Main components of the HSD. Whereas **a**. real photo of the HSD, **b**. double air circulation passes inside the flat plate solar collector.

**Fig. 3:**
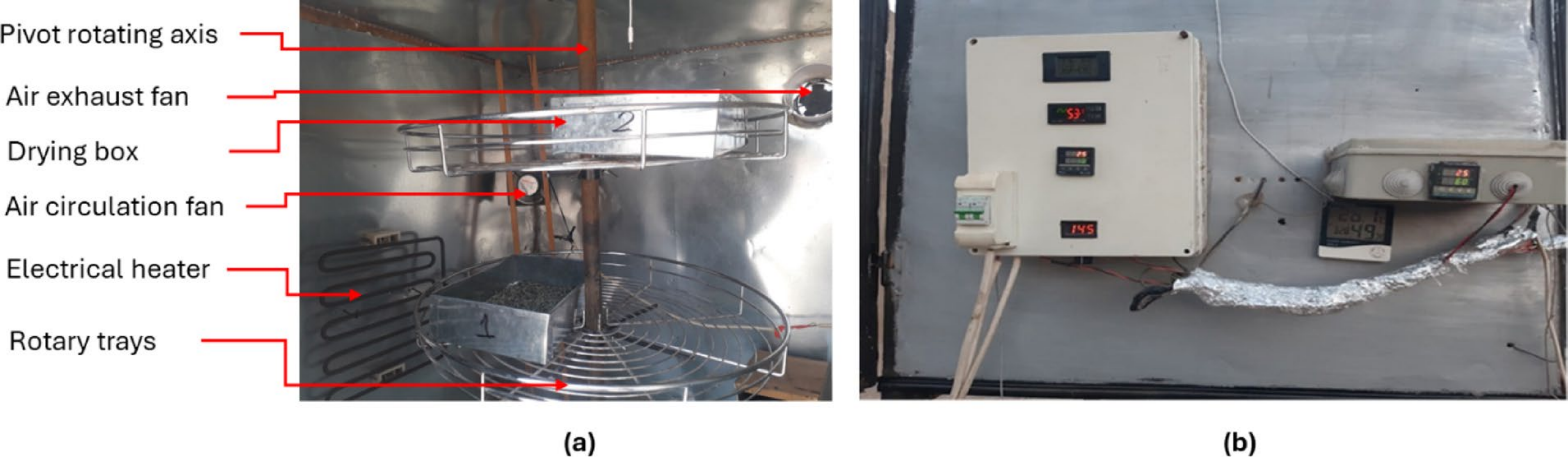
Internal components and control units of the HSD. Whereas, **a**. internal components of the drying room, and **b**. control units for temperature, humidity and electrical heater.

The HSD was created and employed to fulfill the objectives of the current investigation. It was combined with a double air pass flat plate solar collector (DPFPSC) and supplemented by an electric heater to modify the air temperature within the drying chamber. The constructed HSD (Fig. 2) comprises various components, including: 1. DPFPSC: Its dimensions are 4 m in length, 1 m in breadth, and 20 centimeters in depth. It was encased in a glass cover measuring 3 mm in thickness. A corrugated black aluminum plate functions as the absorber plate, while thermal wool with a thickness of 5.0 cm provides insulation. The drying chamber measures 1.0 m in length, breadth, and height. Galvanized steel, measuring 5 mm in thickness, constituted the internal and external walls. The walls were insulated with 2 cm of thermal wool and 3 cm of foam. 4. Drying boxes: Constructed from stainless steel, they measure 30 cm in length, 20 cm in width, and 7 cm in height. Their base is perforated, facilitating the passage of hot air through the items. 5. Rotary trays: The drying chamber contained two rotary trays (Fig. 3), each with a diameter of 60 cm and a height of 3 cm, mounted on a hinged rotary axis. An auxiliary heating system consisting of a 2000 W electric heater was employed alongside solar drying to elevate air temperature and decrease relative humidity within the drying chamber, hence expediting the drying process and minimizing drying duration. 7. Air fans: Two air fans were employed to expel the heated air from the DPFPSC into the drying chamber and to circulate the drying air within the chamber (Model C.C.P. Parm with a flow rate of 6.6 m³/h, 2800 rpm, and 150 W, Italy). 8. Control System: The present study employed two distinct control systems. Two control systems exist. The initial component is a digital controller (model: REX_C100 PID, Yueqing Juzlong Electric Co., Ltd., Yueqing City, Wenzhou, Zhejiang Province, China) that regulates the temperature and humidity within the drying chamber by managing the electrical heater and the airflow fan’s velocity. The secondary system governed the functioning of the air blowers to sustain the requisite temperature and humidity levels within the drying chamber.

The HSD primarily operates by utilizing direct solar thermal energy to heat the drying air. Solar radiation is captured through the double-pass flat plate solar collector (DPFPSC), where a black corrugated aluminum absorber plate converts solar energy into thermal energy. The heated air is then directed into the drying chamber by air fans, facilitating the drying of the product through natural convection and forced air circulation. To ensure consistent drying performance—especially during periods of low solar irradiance, such as cloudy days or late afternoon hours, an auxiliary electric heater (2000 W) is integrated into the system. This heater supplements the solar energy by raising the temperature of the air entering the drying chamber and reducing the relative humidity, which accelerates the drying rate and improves efficiency. Therefore, the primary drying mechanism is solar-based, with electric heating serving as a supportive, secondary source to maintain optimal drying conditions and minimize variability due to fluctuating weather. This hybrid design ensures that the drying process remains efficient, continuous, and less dependent on environmental conditions, offering both energy efficiency and reliability for practical applications.

### Evaluations processes

#### Drying kinetics

##### Moisture content (MC)

Using the procedure outlined by AOAC^62^the fresh pomegranate peels sample was heated to 105 ± 1 °C in an electrical oven for three hours in order to determine the MC of the pomegranate peels under laboratory conditions. The initial MC on a dry basis (d.b.) was then determined using Eq. (1) ^63-65^.

1 $$\:{\mu\:}_{d}=\left[\frac{{W}_{w}-\:{W}_{d}}{{W}_{d}}\right]\times\:100$$

where $\:{\mu\:}_{d}$ is the MC on dry basis, %; $\:{W}_{w}$ is the weight of fresh pomegranate peels sample, g and $\:{W}_{d}$ is the weight of dried pomegranate peel sample, g.

##### Moisture ratio (MR)

The MR was estimated using Eq. (2)^66^.

2 $$\:MR=\frac{{M}_{t}-{M}_{e}}{{M}_{0}-{M}_{e}}$$

where: $\:{M}_{0}$ is the initial MC in %, $\:{M}_{e}$ is the EMC in %, and $\:{M}_{t}$ is the MC at any time in %.

The MR was employed to examine the drying kinetics of pomegranate peels using suitable mathematical models. The value of $\:{M}_{e}$ can be neglected, as it is relatively little compared to the values of $\:{M}_{t}$ and $\:{M}_{0}$, using Eq. (3).

3 $$\:MR=\frac{{M}_{t}}{{M}_{0}}$$

#### Drying constant (*k*)

The drying constant is ascertained using the exponential correlation between LnMR and drying time. Although drying constants are essential for thoroughly characterizing the drying kinetics of dried product^41,67^it is imperative to account for the various transport parameters involved. The drying constant was calculated using Eq. (4).

4 $$\:MR={M}_{0}\text{\:exp}(-k\times\:t)$$

where, t is the time, h.

#### Effective moisture diffusivity (EMD)

Fick’s Second Law of Diffusion (Eq. 5) is a powerful tool for understanding and predicting moisture diffusion behavior in materials. By applying this principle, researchers and engineers can gain valuable insights into EMD, leading to advancements in various fields that rely on controlling moisture transport^68^as follows:

5 $$\:\frac{\partial\:\text{M}\text{C}}{\partial\:\text{t}}={\text{D}}_{\text{e}\text{f}\text{f}}\times\:{\nabla\:}^{2}\text{M}\text{C}$$

Using Eq. (6), the D_eff_ can be calculated,

6 $$\:{\text{MR}} = \:\frac{8}{{\pi ^{2} }} \times \:\sum\limits_{{{\text{n}} = 1}}^{\infty } {\frac{1}{{{\text{n}}^{2} }}{\text{exp}}\left( {\frac{{ - \pi ^{2} \times \:{\text{D}}_{{{\text{eff}}}} \times \:{\text{t}}}}{{4{\text{L}}^{2} }}} \right)}$$

where, n is the term number, t is the time in s, and $\:\text{L}$ is the half slab thickness.

#### Mathematical modelling

The experimental drying data were evaluated through nonlinear least squares regression to fit twelve established thin-layer drying models, as detailed in Table 2. This regression technique minimizes the sum of squared differences between the observed and predicted values, ensuring accurate model fitting. To perform the statistical analysis, specialized software developed by Öksüz and Buzrul^69^was employed. This software facilitated precise comparison among the models by computing key statistical parameters, ultimately aiding in the selection of the most suitable model for describing the drying behavior of the studied product.

**Table 2:** Selected mathematical modeling to demonstrate the drying process of pomegranate peels.

No.	Model name	Model Equation*	Reference
1	Aghbashlo	$\:MR=\text{exp}\left(-\frac{{k}_{1}t}{1+{k}_{2}t}\right)$	^69,70^
2	Henderson - Pabis	$\:MR=a\:\text{e}\text{x}\text{p}\left(-kt\right)$	^71–73^
3	Lewis (Newton)	$\:\text{M}\text{R}=\text{e}\text{x}\text{p}\left(-\text{k}\text{t}\right)$	^71^
4	Logarithmic (Asymptotic)	$\:\text{M}\text{R}=\text{a}\text{*}\text{e}\text{x}\text{p}\left(-\text{k}\text{t}\right)+c$	^71–73^
5	Midilli	$\:\text{M}\text{R}=\text{a}\text{*}\text{e}\text{x}\text{p}\left(-\text{k}{\text{t}}^{n}\right)+bt$	^71–73^
6	Modified Midilli I	$\:\text{M}\text{R}=\text{e}\text{x}\text{p}\left(-\text{k}{\text{t}}^{n}\right)+bt$	^74,75^
7	Modified Midilli II	$\:\text{M}\text{R}=\text{a}\text{*}\text{e}\text{x}\text{p}\left(-\text{k}{\text{t}}^{n}\right)+b$	^74^
8	Modified Page	$\:\text{M}\text{R}=\text{e}\text{x}\text{p}\left(-{\left(\text{k}\text{t}\right)}^{\text{n}}\right)$	^71–73^
9	Page	$\:\text{M}\text{R}=\text{e}\text{x}\text{p}\left(-\text{k}{\text{t}}^{\text{n}}\right)$	^71–73^
10	Wang-Sigh	$\:MR=1+bt+a{t}^{2}$	^71–73^
11	Weibullian	$\:\text{M}\text{R}=\text{e}\text{x}\text{p}\left(-{\left(\frac{t}{\alpha\:}\right)}^{\beta\:}\right)$	^74,75^
12	Weibullian I	$\:\text{M}\text{R}={10}^{-{\left(\frac{t}{\delta\:}\right)}^{n}}$	^74,75^

* k_1,_ k_2_ and k are the drying constants, h^− 1^; t is the drying time, h; a, b,c, n,ɤ, β and δ are the models constants.

#### Thermodynamic properties of pomegranate peels

Thermodynamic properties are essential for understanding and optimizing the drying process of pomegranate peel, as they provide insight into the energy requirements, moisture removal mechanisms, and quality preservation during drying. The main thermodynamic properties studied include enthalpy, entropy, gibbs free energy, activation energy. Where enthalpy changes reflect the total energy required for moisture removal, including both latent heat and binding energy. In pomegranate peel drying, enthalpy values are lower than those for seeds, indicating less energy is needed for peel dehydration. And entropy changes provide information about the disorder or randomness associated with water molecules during drying. Lower entropy in peels compared to seeds suggests a more ordered state of water binding in the peel matrix. A linear relationship between enthalpy and entropy (enthalpy–entropy compensation) indicates that the sorption process is enthalpy-controlled, meaning energy input is the dominant factor in water removal. Additionally, gibbs free energy indicates the spontaneity of the drying process. Positive values suggest that the drying process is non-spontaneous and requires energy input, which is typical for food dehydration. Peels generally show lower Gibbs free energy values than seeds, reflecting easier moisture removal^53,76^. Furthermore, the activation energy is the minimum energy required to initiate moisture diffusion. For pomegranate peel, activation energy values are reported between 10.60 and 22.25 kJ/mol, indicating moderate energy requirements for water removal and supporting the use of moderate drying temperatures for efficiency^53,54,77^. The thermodynamic properties related to the drying process of pomegranate peels were determined by following the methodology described by Jideani and Mpotokwana^78^widely applied to several agricultural products (Eqs. 7–10)

7 $$\:\varDelta\:H=\:{E}_{a}-RT$$

8 $$\:\varDelta\:S=\:R\left(\text{ln}{D}_{0}-\text{ln}\frac{{K}_{B}}{{K}_{P}}-\:\text{ln}T\right)$$

9 $$\:\varDelta\:G=\:\varDelta\:H-T\varDelta\:S$$

10 $$\:{D}_{eff}=\:{D}_{0}{exp}\left(-\frac{{E}_{a}}{RT}\right)$$

where ΔH is the enthalpy (J mol^− 1^), R is the universal gas constant (8.314 J mol^− 1^ K^− 1^), ΔS is the entropy (J mol^− 1^ K^− 1^), K_B_ is the Boltzmann’s constant (1.38 × 10^–23^ J K^− 1^), K_p_ is the Planck’s constant (6.626 × 10^− 34^ J s^− 1^), ΔG is the Gibbs free energy (J mol^− 1^), D_0_ is the pre-exponential factor, and Ea = activation energy (J mol^− 1^).

I. The equations used in drying models and thermodynamic analysis are based on several key assumptions that simplify the complex physical processes involved. Understanding these assumptions is crucial for interpreting results and recognizing the limitations of the models. Common assumptions in drying models:

- Uniform properties: The material being dried is often assumed to have uniform initial MC, temperature, and physical properties throughout the sample. This simplifies calculations but may not reflect real variations within the material during drying^4,79,80^.
- Constant drying conditions: Many models assume constant drying air temperature, velocity, and humidity, even though these can fluctuate in practical settings^79,81,82^.
- Negligible external resistance: It is frequently assumed that the main resistance to moisture movement is internal (within the material), and external resistance (at the surface) is negligible, especially at high air velocities^79,80^.
- Local equilibrium: Some models assume local thermal and/or mass equilibrium between the solid and the drying air, meaning temperature and moisture gradients within the material are ignored or minimized. However, more advanced models may relax this assumption to account for non-equilibrium effects^80,83^.
- Neglecting shrinkage and structural changes: Many drying models do not account for physical changes in the material, such as shrinkage, cracking, or changes in porosity, which can affect drying rates and thermodynamic properties^83,84^.

II. Assumptions in Thermodynamic Analysis.

- Closed or controlled system: Analyses often treat the drying chamber or sample as a closed or well-defined control volume, ignoring interactions with the external environment except for specified inputs and outputs^84,85^.
- Steady-state or quasi-steady-state: Thermodynamic calculations may assume steady-state or quasi-steady-state conditions, where properties change slowly enough to be considered constant over short time intervals^81,84,85^.
- Ideal gas behavior: The drying air is typically assumed to behave as an ideal gas, simplifying calculations of energy and mass transfer^79,86,87^.
- Negligible heat losses: Some models neglect heat losses to the surroundings, assuming all supplied energy is used for moisture removal, which can overestimate efficiency^86–88^.

These assumptions help make drying models and thermodynamic analyses mathematically manageable and useful for engineering design and optimization. However, they may introduce errors when real-world conditions deviate from these simplifications, so results should be interpreted with these limitations in mind.

### Uncertainty analysis

Where the uncertainties in measuring instruments are estimated using Eq. (11)^16^:

11 $$\:{\mathcal{W}}_{r}={\left[{\left(\frac{\partial\:R}{\partial\:{x}_{1}}{\mathcal{W}}_{1}\right)}^{2}+{\left(\frac{\partial\:R}{\partial\:{x}_{2}}{\mathcal{W}}_{2}\right)}^{2}+\dots\:+{\left(\frac{\partial\:R}{\partial\:{x}_{3}}{\mathcal{W}}_{3}\right)}^{2}\right]}^{1/2}$$

The initially evaluated uncertainty of MC, MR and drying rate was about 1.12%. The research also examined measurement errors for temperature, humidity, wind speed, and solar radiation, which were 0.32%, 0.28%, 0.24%, and 0.13%, respectively. Upon evaluating these factors, the comprehensive assessment of HSD efficiency was estimated to possess a cumulative uncertainty of around ± 2%. This signifies that, notwithstanding the intrinsic measurement uncertainties, the sun drying system continues to be dependable and efficient in its functioning. The accumulation of imprecision highlights the system’s resilience in practical scenarios.

### Statistical analysis

The coefficient of determination (*R*^2^), the adjusted coefficient of determination ($\:{R}_{adj.}^{2}$) and root mean squared error (RMSE) are fundamental statistical criteria for selecting the optimal model to characterize the drying curves. The optimal model was identified based on the criterion of minimal RMSE values and maximal *R*^2^ and $\:{R}_{adj.}^{2}$^84,85^. The following Eqs. (12–14) can be employed to calculate these parameters.

12 $$\:{R}^{2}=1-\frac{\sum\:_{i=1}^{N}{{(MR}_{pre,\:i}-{MR}_{obs,\:i})}^{2}}{\sum\:_{i=1}^{N}{{(\stackrel{-}{M}R}_{pre}-{MR}_{obs,\:i})}^{2}}$$

13 $$\:{R}_{adj.}^{2}=1-\left(1-{R}^{2}\right)*\frac{N-1}{N-n}$$

14 $$\:RMSE=\sqrt{\frac{1}{N}{\sum\:}_{i=1}^{N}{{(MR}_{pre,\:i}-{MR}_{obs,\:i})}^{2}}$$

where, MR_exp, i_ is experimental MR; MR_pre, i_ is predicted MR; N is number of observations; n is number of constants.

## Results and discussions

### Moisture content

The impact of drying methods, layer thicknesses and drying temperatures on MC of pomegranate peels are shown in Tables S1 and S2 in supplementary materials. The average initial MC of pomegranate peels is 325.53% (d.b.). The drying process using OD led to a decrease in the MC of all pomegranate peel samples from 325.53% to average MC of 2.80, 2.32, and 2.15%, at drying temperatures of 50, 60, and 70 °C, respectively. Additionally, the average MC of all pomegranate peel samples dried using HSD were 3.01, 2.64, and 2.92%, at drying temperatures of 50, 60, and 70 °C, respectively. On the other hand, the drying process using OD led to a decrease in the MC of all pomegranate peel samples from 325.53% to average MC of 2.82, 2.28, and 2.18%, at layer thicknesses of 1, 2, and 3 cm, respectively. Furthermore, drying of pomegranate peel using HSD led to decrease the MC up to 3.08, 3.16, and 2.33%, at layer thicknesses of 1, 2, and 3 cm, respectively.

### MR (MR)

**Fig. 4:**
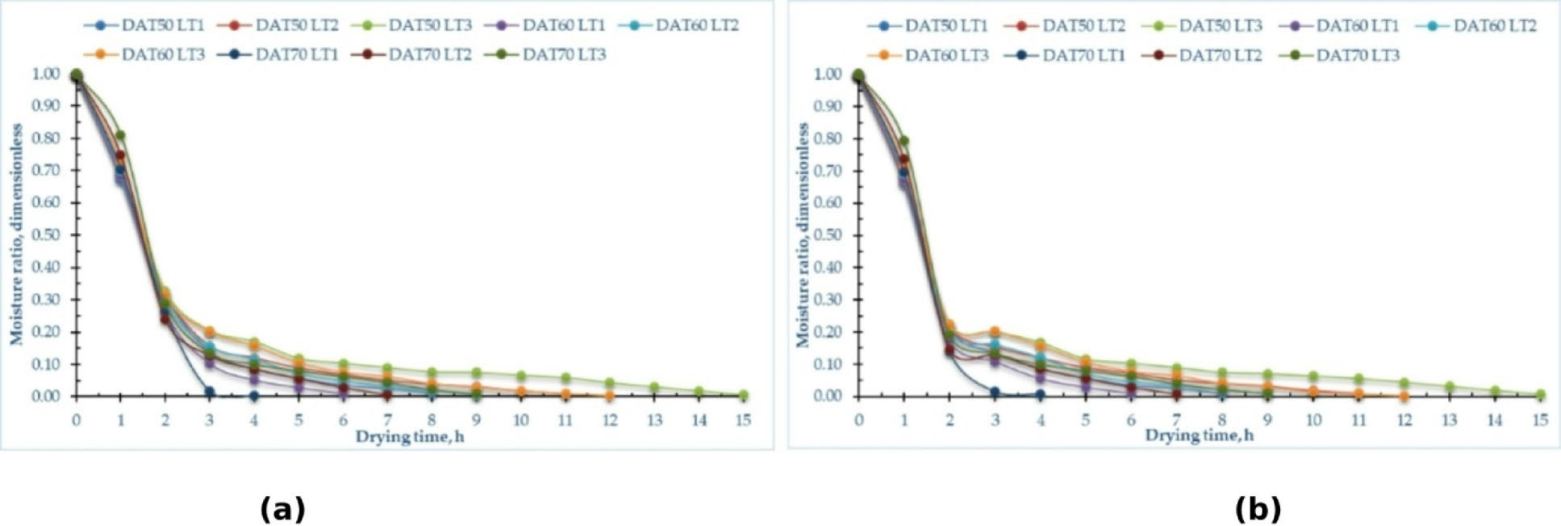
MR on dry basis of pomegranate peels at different drying temperatures and layers thicknesses, (**a**) OD, and (**b**) HSD.

Figure 4 and Tables S3 and S4 in supplementary materials illustrate the variation of MR relative to drying time for pomegranate peels at various drying temperatures and layer thicknesses across both drying processes. Furthermore, Fig. 4 illustrates the variations in the drying process at three distinct temperatures: 50, 60, and 70 °C, as well as three different layer thicknesses of 1, 2, and 3 cm. The drying time required to attain the equilibrium MC ranged from 4 to 15 h, depending on the drying temperature and layer thickness. Furthermore, Fig. 4 illustrates that increasing the air temperature from 50 to 70 °C results in a decrease in the drying time of the finished product by about 100%, 57%, and 67% for layer thicknesses of 1 cm, 2 cm, and 3 cm, respectively, across both drying techniques. This observation corresponds with the results presented by Beigi^88^ and Kaleta et al.^89^which indicated that increasing the air temperature from 50 to 60 °C during the drying process improved mass transfer, reduced process length, and minimized energy consumption. Also, Kara and Doymaz’s^90^ indicated that elevated air temperatures resulted in reduced drying times. Furthermore, Beigi^88^ noted that an increase in air temperature within the measured range resulted in a proportional increase in the amount of moisture extracted from the product. The MR during drying is substantially affected by air temperature and layer thickness. Elevated air temperatures accelerate the drying rate by augmenting the kinetic energy of water molecules, resulting in a more pronounced reduction in MC over time. For example, drying pomegranate peels demonstrated that elevating the temperature from 50 °C to 70 °C leads to a more rapid decrease in MR, attributable to heightened vapor pressure differentials and accelerated moisture evaporation. Conversely, layer thickness influences the diffusion path length for moisture extraction. Thinner layers (1 cm) facilitate expedited and uniform drying, resulting in a swift decrease in MC, but thicker layers (3 cm) augment internal barrier to moisture transfer, so decelerating the drying process.

Table 3 presents the drying coefficient (k) and determination coefficient (R²) for pomegranate peels at various drying temperatures and layer thicknesses for both OD and HSD methods. The tabulated data indicated that the (k) increases with the rise in drying air temperature in both drying methods. Higher drying temperatures result in increased vapor pressure of water within pomegranate peels. The elevated vapor pressure generates a greater impetus for water molecules to evaporate from the surface. Furthermore, augment the kinetic energy of the water molecules contained in the pomegranate peels. The augmented kinetic energy leads to accelerated passage of water molecules from the interior to the surface, facilitating evaporation. Conversely, the data indicated that the drying coefficient often diminishes as layer thickness increases. As the layer thickness grows, the distance water molecules must traverse from the core of the pomegranate peels to the surface for evaporation likewise increases. The extended diffusion pathway results in heightened resistance to water movement and a reduced drying rate. Moreover, thicker layers of pomegranate peels possess a reduced surface area to volume ratio in comparison to thinner layers. This indicates that a reduced fraction of the pomegranate peels is directly subjected to the drying conditions, hence constraining the rate of moisture extraction. Moreover, with thicker layers of pomegranate peels, temperature differences may arise during the drying process. The external surface of pomegranate peels may be considerably warmer than the inner core, resulting in uneven drying and potentially obstructing moisture extraction from the interior. These findings aligned closely with the analogous pattern reported in the drying rate data. Doymaz^91^Kaleta et al.^92^and Meziane^93^ made analogous observations. The coefficient of determination indicated that the tabulated data exhibited a direct correlation with layer thickness and an inverse correlation with drying temperature.

**Table 3:** Drying coefficient and determination coefficient of pomegranate peels for OD and HSD.

Drying system	Coefficient	DAT50			DAT60			DAT70		
		LT1	LT2	LT3	LT1	LT2	LT3	LT1	LT2	LT3
OD	**k**	-0.554	-0.394	-0.257	-0.778	-0.509	-0.412	-1.428	-0.689	-0.481
	**R** ^**2**^	0.989	0.973	0.921	0.992	0.985	0.982	0.928	0.963	0.965
HSD	**k**	-0.544	-0.368	-0.248	-0.766	-0.468	-0.417	-1.329	-0.644	-0.461
	**R** ^**2**^	0.981	0.962	0.914	0.986	0.977	0.966	0.95	0.949	0.953

### Effective moisture diffusivity

**Fig. 5:**
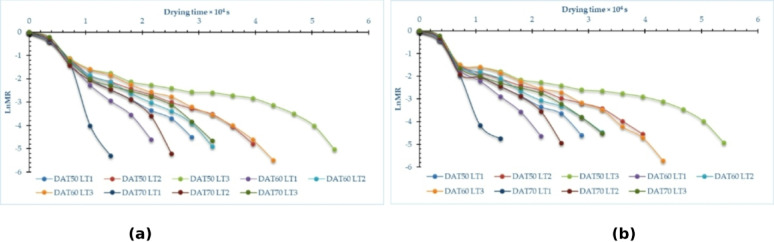
Relation between LnMR and drying time of pomegranate peels at different drying temperatures and layers thicknesses, (**a**) OD, and (**b**) HSD.

Figure 5 and Tables S5 and S6 in supplementary materials illustrate the correlation between LnMR and the drying time of pomegranate peels for OA and DDS across various layer thicknesses and drying temperatures. A linear correlation between drying time and ln (MR) was seen in both drying methods. The principal experimental parameter used for simulating drying processes is the decrease in sample weight, measured as the ratio of the water content at a specific time (t) to the initial MC^94^.

**Fig. 6:**
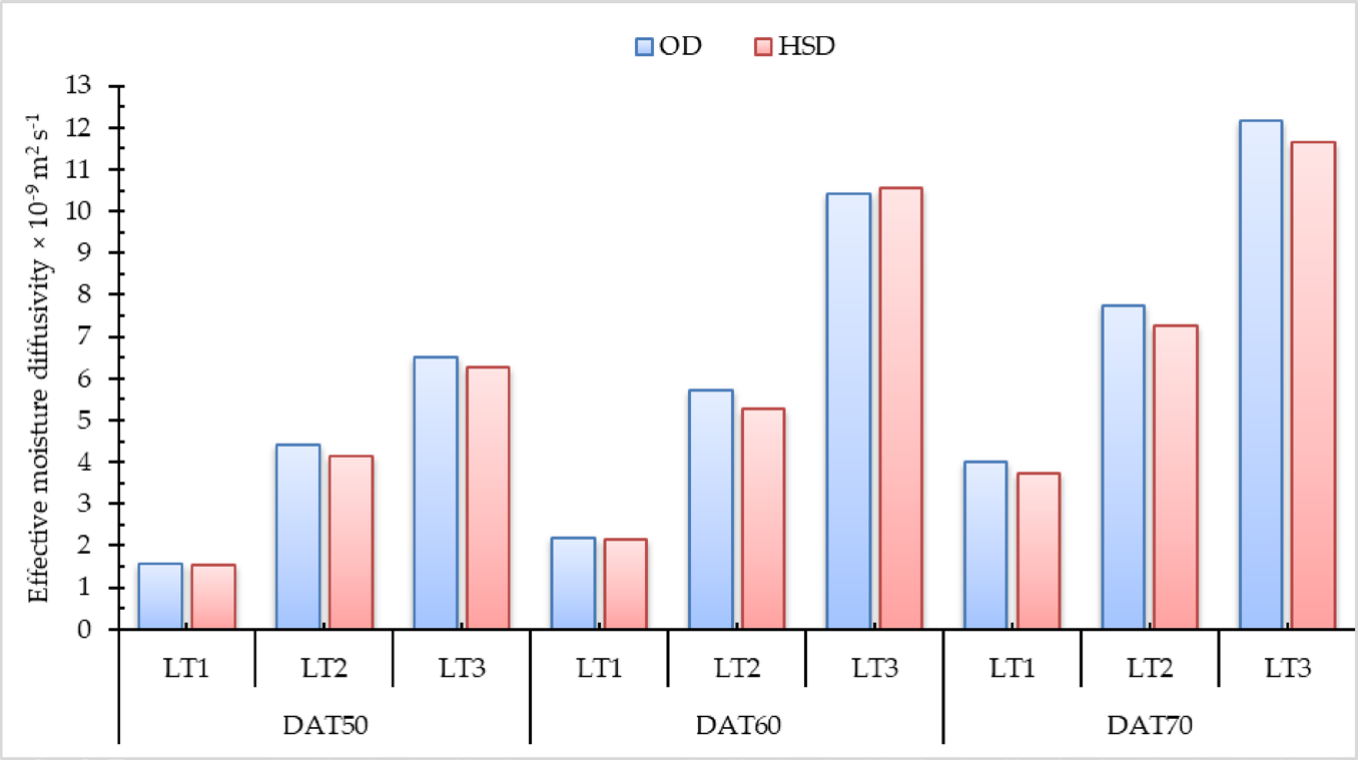
EMD for OD and HSD under different drying air temperatures and different layers thicknesses.

The results illustrated in Fig. 6 reveal that the drying time is predominantly controlled by internal mass transfer resistance, which is influenced by the presence of a falling-rate drying period. Accordingly, the EMD values for the drying experiments under various conditions were calculated using Fick’s second law of diffusion. Similar patterns were observed across the same thin-layer thickness, demonstrating that the water diffusion coefficient increases as temperature rises. Specifically, at a drying temperature of 70 °C, the EMD values for layer thicknesses of 1.0, 2.0, and 3.0 cm were 4.02, 7.75, and 12.17 × 10⁻⁹ m² s⁻¹, respectively, for the OD system, and 3.74, 7.25, and 11.66 × 10⁻⁹ m² s⁻¹, respectively, for the HSD system. The highest EMD recorded in this study occurred at 70 °C with a 3 cm layer thickness, emphasizing that air temperature is a crucial factor affecting EMD. As temperature increases, the kinetic energy of water molecules also increases, which accelerates moisture migration and leads to higher EMD values^4,93,95,96^. Conversely, layer thickness has a significant impact on the moisture transfer pathway; thicker layers generally reduce EMD because they introduce more resistance to moisture movement by lengthening the diffusion path^4,97^. The EMD values obtained in this investigation are consistent with previous findings. For instance, Mphahlele et al.^59^ reported EMD values for pomegranate peel ranging from 4.05 × 10⁻¹⁰ to 8.10 × 10⁻¹⁰ m² s⁻¹ when drying at air temperatures of 40, 50, and 60 °C with a constant air velocity of 1.0 m/s. Similarly, Doymaz^4^ studied the drying of pomegranate peels using a cabinet dryer at an air velocity of 2.0 m/s and an initial layer thickness of 2.8 cm, with air temperatures of 50, 60, and 70 °C. In that study, the EMD ranged from 4.02 to 5.31 × 10⁻⁹ m² s⁻¹ across the temperature range examined.

### Activation energy

**Fig. 7:**
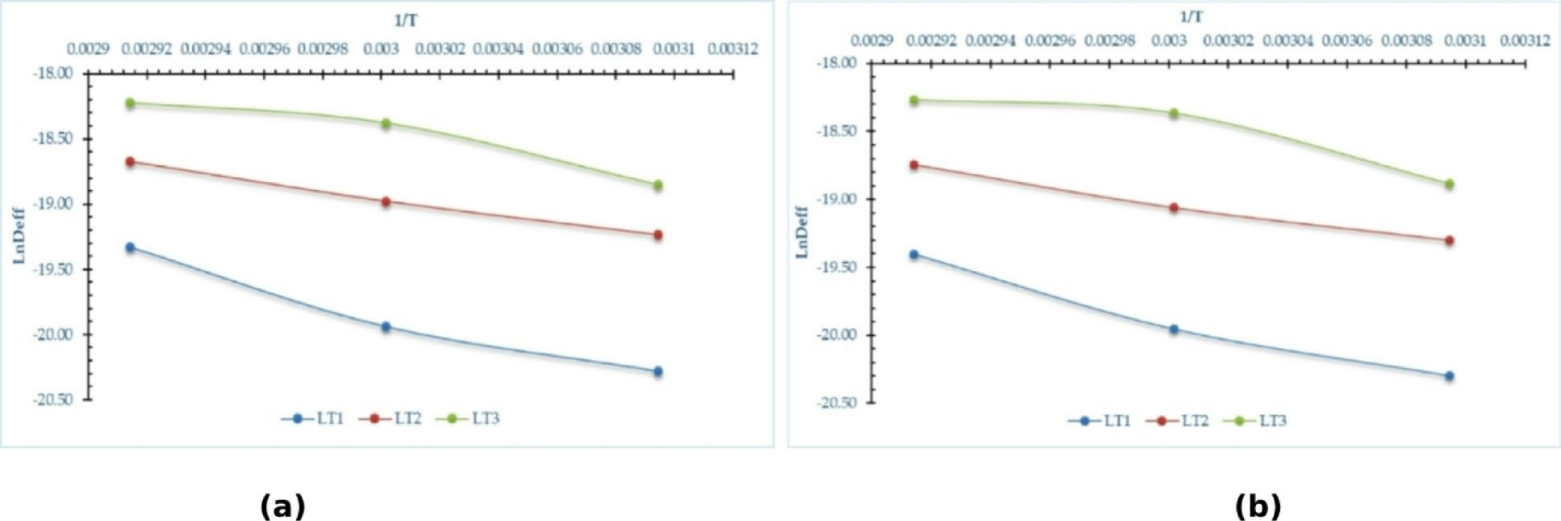
LnD_eff_ with invers absolute temperature of pomegranate peels at different drying temperatures and layers thicknesses, (**a**) OD, and (**b**) HSD.

The activation energy can be computed using a method analogous to that employed for determining EMD. Utilized the EMD values collected previously to construct linear correlations with the inverse of the absolute drying temperature data, as illustrated in Fig. 7.

**Fig. 8:**
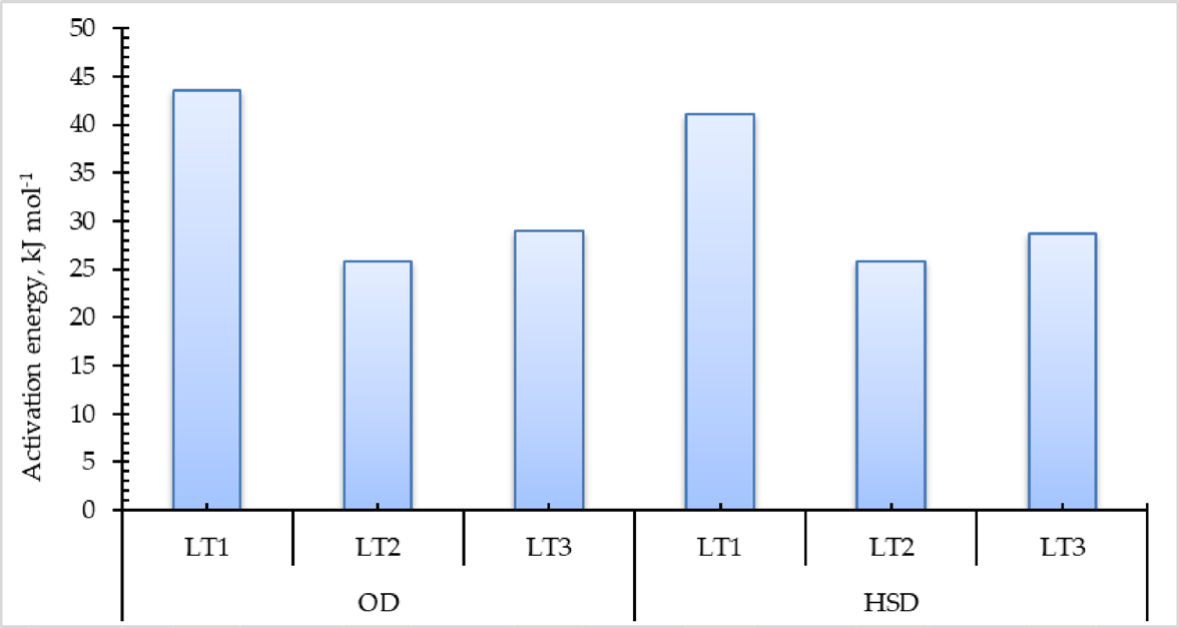
Activation energy of pomegranate peels for OD and HSD under different drying air temperatures and different layers thicknesses.

Figure 8 shows the activation energy of different pomegranate peels samples, where it was calculated to find the minimum energy at which the drying occurs. Consequently, the activation energy is ascertained by calculating the slope of the linear correlations depicted in Fig. 7. Figure 8 illustrates that in this investigation, the activation energy values for thicknesses of 1.0, 2.0, and 3.0 cm were 43.55, 25.76, and 29.06 kJ mol⁻¹ for the OD, and 41.09, 25.82, and 28.72 kJ mol⁻¹ for the HSD. In addition, these results are in close agreement with those reported by Mphahlele et al.^59^who investigated the drying of pomegranate peels at air temperatures of 40 °C, 50 °C, and 60 °C, using a constant air velocity of 1.0 m/s. Their study revealed an average activation energy of 22.25 kJ mol⁻¹. Similarly, Doymaz^4^ conducted drying experiments on pomegranate peels using a cabinet dryer at a constant air velocity of 2.0 m/s and an initial layer thickness of 2.8 cm, with drying air temperatures of 50 °C, 60 °C, and 70 °C. In that case, the activation energy was determined to be 12.72 kJ mol⁻¹.

### Mathematical **modeling**

**Fig. 9:**
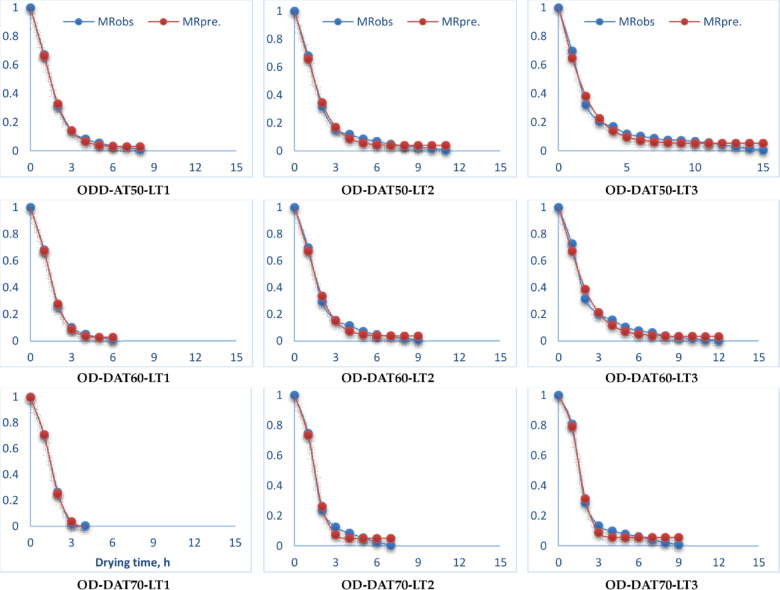
Observed and predicted MR for OD at the best mathematical model (Modified Midilli II).

**Fig. 10:**
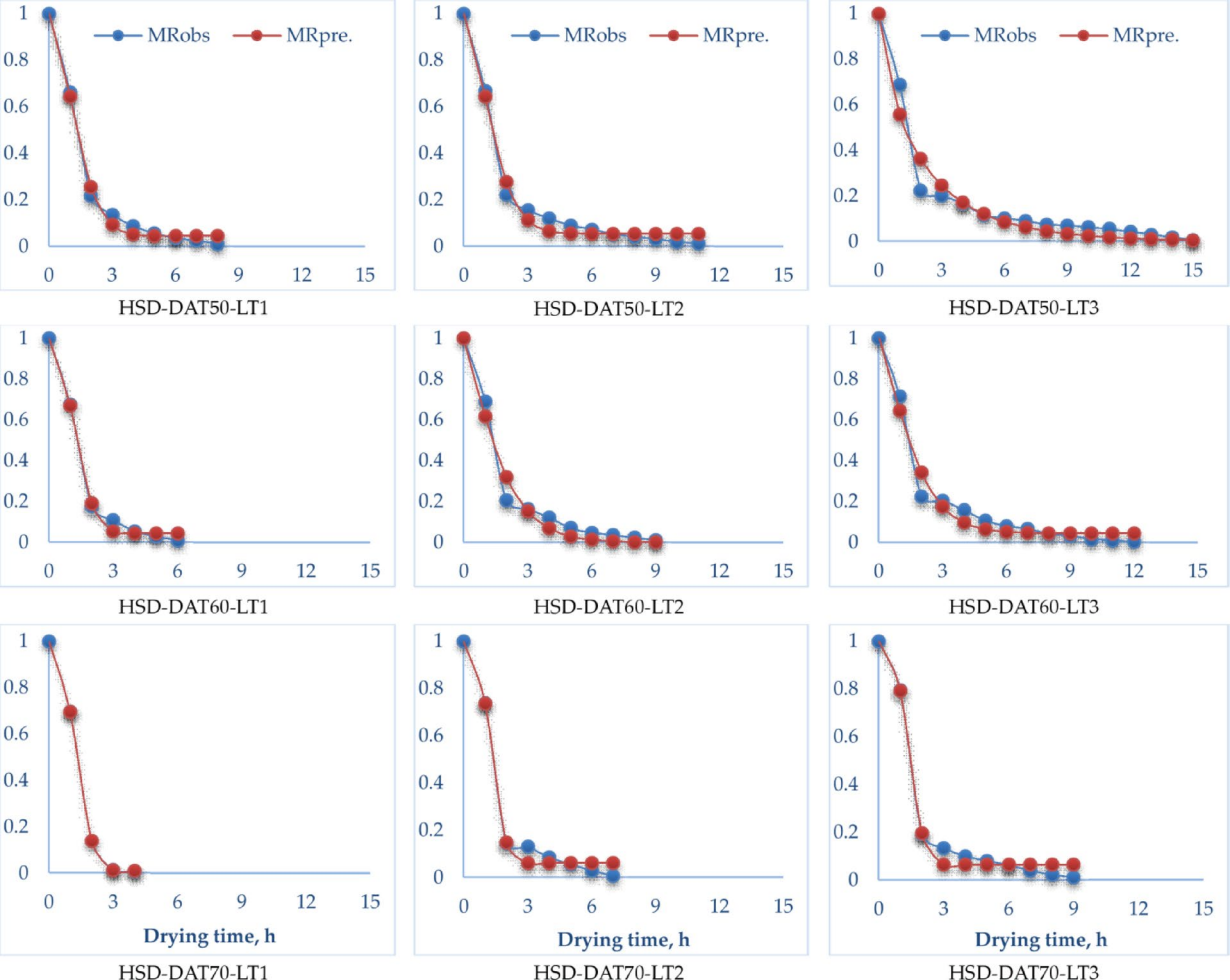
Observed and predicted MR for HSD at the best mathematical model (Modified Midilli II).

Table 4 shows the mathematical model constants and statistical parameters of OD and HSD for pomegranate peels under different drying air temperatures and layer thicknesses. Twelve mathematical models, detailed in Table 2, were utilized to examine the drying kinetics of pomegranate peels. Initially, the MC data of the dried samples were recorded at different drying air temperatures and layer thicknesses. Subsequently, these MC values were transformed into the MR using Eq. 6, after which the twelve mathematical models were applied to perform curve fitting on the experimental data. Selecting a suitable mathematical model is crucial for accurately predicting the drying behavior of various products^98^. However, the selection of the most appropriate model for describing the drying kinetics of pomegranate peels should not be based solely on the number of constants in the model. Instead, the process must be guided by statistical criteria that have been validated in the literature as effective indicators for model selection. Therefore, it is essential to rigorously assess these statistical parameters to ensure that the choice of mathematical model is informed by reliable data and sound analytical reasoning. The results of the statistical analysis, shown in Table 4, indicate that all tested drying models exhibited generally high values of *R*^2^and $\:{R}_{adj.}^{2}$, along with low values of the RMSE. These statistical measures are widely recognized and used by researchers to evaluate the goodness-of-fit of drying models. Numerous studies have confirmed that the best model for describing thin-layer drying behavior is the one characterized by the highest *R*^2^and $\:{R}_{adj.}^{2}\:$values, as well as the lowest RMSE^84,85^. Furthermore, all twelve fundamental mathematical models were employed to simulate the drying kinetics of pomegranate peels under the examined conditions. Among them, the Modified Midilli (II) model demonstrated the best fit and most accurately described the drying kinetics of pomegranate peels for both drying techniques—OD and HSD—as reflected in Table 4; Figs. 9 and 10.

**Table 4:** Mathematical models’ constants values and goodness of fit indices results of OD and HSD for pomegranate peels under different drying temperatures (DAT) and different layers thicknesses (LT).

MMs	DAT, °C	LT, cm	Parameters	Oven drying (OD)							Parameters	Hybrid solar drying (HSD)						
				Models’ constants values				Goodness of fit indices				Models’ constants values				Goodness of fit indices		
				Values	S.E.	p-value	Sign. – Insign.	RMSE	R^2^	R^2^_adj_.		Values	S.E.	p-value	Sign. – Insign.	RMSE	R^2^	R^2^_adj_.
**Aghbashlo**	**50**	**1**	**k**_**1**_	0.0400	0.0547	0.4884	**InSign.**	0.495552	-0.762515	-1.014303	**k**_**1**_	0.0400	0.0568	0.5040	**InSign.**	0.514463	-0.923595	-1.198394
			**k**_**2**_	-0.1250	0.0506	0.0429	Sign.				**k**_**2**_	-0.1250	0.0526	0.0490	Sign.			
		**2**	**k**_**1**_	0.5119	0.0559	3.53*10^− 6^	Sign.	0.037373	0.986951	0.985646	**k**_**1**_	0.6024	0.0905	5.67*10^− 5^	Sign.	0.050645	0.975348	0.972883
			**k**_**2**_	-0.0009	0.0324	0.9788	**InSign.**				**k**_**2**_	0.0324	0.0498	0.5306	**InSign.**			
		**3**	**k**_**1**_	0.0400	0.0346	0.2668	**InSign.**	0.428267	-1.285235	-1.448466	**k**_**1**_	0.0400	0.0356	0.2801	**InSign.**	0.440892	-1.472200	-1.648786
			**k**_**2**_	-0.0667	0.0306	0.0470	Sign.				**k**_**2**_	-0.0667	0.0315	0.0527	**InSign.**			
	**60**	**1**	**k**_**1**_	0.0400	0.0864	0.6629	**InSign.**	0.530652	-0.571124	-0.885348	**k**_**1**_	0.0400	0.0898	0.6745	**InSign.**	0.551200	-0.684874	-1.021849
			**k**_**2**_	-0.1667	0.0975	0.1479	**InSign.**				**k**_**2**_	-0.1667	0.1012	0.1606	**InSign.**			
		**2**	**k**_**1**_	0.4711	0.0647	8.52*10^− 5^	Sign.	0.044056	0.984822	0.982925	**k**_**1**_	0.5429	0.1047	0.0008	Sign.	0.061347	0.970158	0.966428
			**k**_**2**_	-0.0419	0.0401	0.3263	**InSign.**				**k**_**2**_	-0.0171	0.0622	0.7898	**InSign.**			
		**3**	**k**_**1**_	0.4762	0.0559	3.57*10^− 5^	Sign.	0.042455	0.982600	0.981018	**k**_**1**_	0.5503	0.0882	6.34*10^− 5^	Sign.	0.057034	0.967820	0.964894
			**k**_**2**_	0.0056	0.0321	0.8651	**InSign.**				**k**_**2**_	0.0322	0.0485	0.5199	**InSign.**			
	**70**	**1**	**k**_**1**_	0.0400	0.2460	0.8812	**InSign.**	0.617217	-0.478052	-0.970736	**k**_**1**_	0.0400	0.2630	0.8888	**InSign.**	0.659794	-0.608543	-1.144724
			**k**_**2**_	-0.2500	0.5640	0.6876	**InSign.**				**k**_**2**_	-0.2500	0.6029	0.7062	**InSign.**			
		**2**	**k**_**1**_	0.0400	0.0652	0.5619	**InSign.**	0.509228	-0.572137	-0.834159	**k**_**1**_	0.0400	0.0678	0.5767	**InSign.**	0.529635	-0.695068	-0.977579
			**k**_**2**_	-0.1429	0.0632	0.0646	**InSign.**				**k**_**2**_	-0.1429	0.0658	0.0728	**InSign.**			
		**3**	**k**_**1**_	0.3710	0.0944	0.0044	Sign.	0.076155	0.958933	0.953799	**k**_**1**_	0.4166	0.1301	0.0126	Sign.	0.090226	0.941558	0.934253
			**k**_**2**_	-0.1111	0.0652	0.1266	**InSign.**				**k**_**2**_	-0.1111	0.0886	0.2451	**InSign.**			
**Henderson - Pabis**	**50**	**1**	**k**	0.5651	0.0391	1.80*10^− 6^	Sign.	0.041041	0.987911	0.986184	**k**	0.6157	0.0572	1.31*10^− 5^	Sign.	0.053270	0.979376	0.976430
			**a**	1.0316	0.0389	2.76*10^− 8^	Sign.				**a**	1.0289	0.0510	1.83*10^− 7^	Sign.			
		**2**	**k**	0.5236	0.0316	1.35*10^− 8^	Sign.	0.036615	0.987475	0.986222	**k**	0.5679	0.0496	4.57*10^− 7^	Sign.	0.051218	0.974788	0.972266
			**a**	1.0222	0.0343	4.22*10^− 11^	Sign.				**a**	1.0185	0.0485	1.33*10^− 9^	Sign.			
		**3**	**k**	0.4487	0.0335	2.24*10^− 9^	Sign.	0.046885	0.972612	0.970656	**k**	0.4928	0.0493	9.43*10^− 8^	Sign.	0.060705	0.953132	0.949785
			**a**	1.0000	0.0428	1.30*10^− 12^	Sign.				**a**	0.9999	0.0563	5.37*10^− 11^	Sign.			
	**60**	**1**	**k**	0.6213	0.0742	0.0004	Sign.	0.068732	0.973642	0.968371	**k**	0.6662	0.0964	0.0010	Sign.	0.081259	0.963382	0.956058
			**a**	1.0425	0.0659	1.83*10^− 5^	Sign.				**a**	1.0407	0.0784	4.35*10^− 5^	Sign.			
		**2**	**k**	0.5425	0.0404	9.00*10^− 7^	Sign.	0.044870	0.984256	0.982288	**k**	0.5803	0.0600	1.09*10^− 5^	Sign.	0.060684	0.970799	0.967148
			**a**	1.0316	0.0422	8.44*10^− 9^	Sign.				**a**	1.0283	0.0576	9.98*10^− 8^	Sign.			
		**3**	**k**	0.4793	0.0319	1.10*10^− 9^	Sign.	0.041740	0.983182	0.981653	**k**	0.5140	0.0490	4.55*10^− 7^	Sign.	0.057950	0.966778	0.963758
			**a**	1.0247	0.0386	2.48*10^− 11^	Sign.				**a**	1.0186	0.0541	1.02*10^− 9^	Sign.			
	**70**	**1**	**k**	0.6602	0.1540	0.0233	Sign.	0.129041	0.935395	0.913860	**k**	0.7332	0.1957	0.0332	Sign.	0.143645	0.923758	0.898344
			**a**	1.0550	0.1249	0.0035	Sign.				**a**	1.0537	0.1401	0.0049	Sign.			
		**2**	**k**	0.5862	0.0802	0.0003	Sign.	0.081556	0.959675	0.952954	**k**	0.6304	0.1070	0.0011	Sign.	0.098245	0.941675	0.931955
			**a**	1.0521	0.0776	9.99*10^− 6^	Sign.				**a**	1.0488	0.0943	3.14*10^− 5^	Sign.			
		**3**	**k**	0.5294	0.0684	5.52*10^− 5^	Sign.	0.080940	0.953609	0.947810	**k**	0.5700	0.0867	0.0002	Sign.	0.092303	0.938836	0.931191
			**a**	1.0626	0.0759	6.60*10^− 7^	Sign.				**a**	1.0566	0.0875	2.04*10^− 6^	Sign.			
**Lewis (Newton)**	**50**	**1**	**k**	0.5504	0.0330	1.69*10^− 7^	Sign.	0.040203	0.986742	0.986742	**k**	0.6009	0.0477	1.49*10^− 6^	Sign.	0.050978	0.978414	0.978414
		**2**	**k**	0.5131	0.0263	6.86*10^− 10^	Sign.	0.035635	0.986950	0.986950	**k**	0.5582	0.0411	3.27*10^− 8^	Sign.	0.049185	0.974425	0.974425
		**3**	**k**	0.4487	0.0273	5.21*10^− 11^	Sign.	0.045295	0.972612	0.972612	**k**	0.4929	0.0407	3.77*10^− 9^	Sign.	0.058647	0.953132	0.953132
	**60**	**1**	**k**	0.6011	0.0617	6.71*10^− 5^	Sign.	0.065401	0.971362	0.971362	**k**	0.6457	0.0799	0.0002	Sign.	0.076209	0.961351	0.961351
		**2**	**k**	0.5278	0.0337	7.75*10^− 8^	Sign.	0.043779	0.983139	0.983139	**k**	0.5661	0.0497	1.19*10^− 6^	Sign.	0.058073	0.969915	0.969915
		**3**	**k**	0.4684	0.0261	5.02*10^− 10^	Sign.	0.040702	0.982553	0.982553	**k**	0.5049	0.0401	2.83*10^− 8^	Sign.	0.055774	0.966429	0.966429
	**70**	**1**	**k**	0.6338	0.1218	0.0065	Sign.	0.115486	0.931007	0.931007	**k**	0.7063	0.1562	0.0106	Sign.	0.127550	0.919848	0.919848
		**2**	**k**	0.5624	0.0666	6.47*10^− 5^	Sign.	0.078389	0.956536	0.956536	**k**	0.6065	0.0885	0.0002	Sign.	0.093028	0.938989	0.938989
		**3**	**k**	0.5029	0.0570	1.00*10^− 5^	Sign.	0.079585	0.949544	0.949544	**k**	0.5439	0.0719	3.46*10^− 5^	Sign.	0.089330	0.935553	0.935553
**Logarithmic (Asymptotic)**	**50**	**1**	**k**	0.5426	0.0613	0.0001	Sign.	0.043367	0.988430	0.984573	**k**	0.6151	0.0903	0.0005	Sign.	0.057538	0.979376	0.972502
			**a**	1.0431	0.0462	4.96*10^− 7^	Sign.				**a**	1.0292	0.0610	2.77*10^− 6^	Sign.			
			**c**	-0.0147	0.0299	0.6413	**InSign.**				**c**	-0.0004	0.0354	0.9924	**InSign.**			
		**2**	**k**	0.5500	0.0457	7.55*10^− 7^	Sign.	0.036892	0.988556	0.986013	**k**	0.6186	0.0704	1.04*10^− 5^	Sign.	0.050080	0.978306	0.973486
			**a**	1.0113	0.0367	5.27*10^− 10^	Sign.				**a**	1.0001	0.0504	9.66*10^− 9^	Sign.			
			**c**	0.0157	0.0170	0.3804	**InSign.**				**c**	0.0264	0.0216	0.2521	**InSign.**			
		**3**	**k**	0.5285	0.0387	4.33*10^− 9^	Sign.	0.034913	0.985898	0.983728	**k**	0.5919	0.0626	3.42*10^− 7^	Sign.	0.049583	0.970966	0.966499
			**a**	0.9765	0.0337	3.38*10^− 13^	Sign.				**a**	0.9734	0.0486	3.70*10^− 11^	Sign.			
			**c**	0.0442	0.0121	0.0030	Sign.				**c**	0.0485	0.0165	0.0114	Sign.			
	**60**	**1**	**k**	0.5307	0.1143	0.0097	Sign.	0.068284	0.979188	0.968782	**k**	0.5971	0.1562	0.0187	Sign.	0.086981	0.966435	0.949652
			**a**	1.0992	0.0875	0.0002	Sign.				**a**	1.0786	0.1056	0.0005	Sign.			
			**c**	-0.0661	0.0752	0.4288	**InSign.**				**c**	-0.0441	0.0826	0.6213	**InSign.**			
		**2**	**k**	0.5410	0.0635	6.10*10^− 5^	Sign.	0.047964	0.984259	0.979761	**k**	0.6004	0.0939	0.0004	Sign.	0.064384	0.971238	0.963021
			**a**	1.0323	0.0493	1.43*10^− 7^	Sign.				**a**	1.0198	0.0664	1.20*10^− 6^	Sign.			
			**c**	-0.0009	0.0284	0.9748	**InSign.**				**c**	0.0113	0.0350	0.7553	**InSign.**			
		**3**	**k**	0.5008	0.0465	8.07*10^− 7^	Sign.	0.042707	0.983993	0.980792	**k**	0.5542	0.0711	1.47*10^− 5^	Sign.	0.058481	0.969242	0.963090
			**a**	1.0159	0.0416	3.03*10^− 10^	Sign.				**a**	1.0043	0.0577	8.25*10^− 9^	Sign.			
			**c**	0.0137	0.0190	0.4896	**InSign.**				**c**	0.0224	0.0245	0.3814	**InSign.**			
	**70**	**1**	**k**	0.3456	0.2213	0.2588	**InSign.**	0.104529	0.971738	0.943477	**k**	0.4613	0.3147	0.2803	**InSign.**	0.141768	0.950492	0.900983
			**a**	1.4516	0.4784	0.0936	**InSign.**				**a**	1.3011	0.3910	0.0797	**InSign.**			
			**c**	-0.4188	0.5052	0.4943	**InSign.**				**c**	-0.2620	0.4089	0.5874	**InSign.**			
		**2**	**k**	0.5361	0.1299	0.0091	Sign.	0.086894	0.961853	0.946594	**k**	0.6021	0.1742	0.0181	Sign.	0.107021	0.942325	0.919255
			**a**	1.0814	0.0988	0.0001	Sign.				**a**	1.0635	0.1189	0.0003	Sign.			
			**c**	-0.0355	0.0739	0.6513	**InSign.**				**c**	-0.0176	0.0802	0.8347	**InSign.**			
		**3**	**k**	0.5201	0.1074	0.0019	Sign.	0.086419	0.953726	0.940505	**k**	0.5774	0.1348	0.0036	Sign.	0.098623	0.938902	0.921446
			**a**	1.0677	0.0889	6.32*10^− 6^	Sign.				**a**	1.0530	0.1016	1.69*10^− 5^	Sign.			
			**c**	-0.0067	0.0530	0.9035	**InSign.**				**c**	0.0046	0.0553	0.9356	**InSign.**			
**Midilli**	**50**	**1**	**k**	0.4260	0.0313	3.82*10^− 5^	Sign.	0.018927	0.998164	0.997062	**k**	0.4757	0.0753	0.0015	Sign.	0.042401	0.990667	0.985067
			**a**	1.0036	0.0188	4.39*10^− 8^	Sign.				**a**	1.0073	0.0423	2.42*10^− 6^	Sign.			
			**b**	0.0038	0.0015	0.0548	**InSign.**				**b**	0.0052	0.0032	0.1650	**InSign.**			
			**n**	1.4071	0.0847	1.45*10^− 5^	Sign.				**n**	1.4934	0.2093	0.0008	Sign.			
		**2**	**k**	0.4485	0.0506	2.06*10^− 5^	Sign.	0.031062	0.992789	0.990084	**k**	0.5063	0.0857	0.0004	Sign.	0.048946	0.981580	0.974673
			**a**	1.0085	0.0309	8.43*10^− 10^	Sign.				**a**	1.0116	0.0488	3.06*10^− 8^	Sign.			
			**b**	0.0035	0.0015	0.0533	**InSign.**				**b**	0.0043	0.0023	0.1006	**InSign.**			
			**n**	1.2442	0.1146	4.60*10^− 6^	Sign.				**n**	1.2564	0.1906	0.0002	Sign.			
		**3**	**k**	0.4864	0.0630	5.38*10^− 6^	Sign.	0.039824	0.983063	0.978829	**k**	0.5484	0.0939	7.95*10^− 5^	Sign.	0.055381	0.966566	0.958207
			**a**	1.0141	0.0396	7.64*10^− 12^	Sign.				**a**	1.0159	0.0552	3.65*10^− 10^	Sign.			
			**b**	0.0033	0.0013	0.0282	Sign.				**b**	0.0034	0.0018	0.0799	**InSign.**			
			**n**	0.9921	0.1080	8.88*10^− 7^	Sign.				**n**	0.9596	0.1510	3.63*10^− 5^	Sign.			
	**60**	**1**	**k**	0.4047	0.0348	0.0014	Sign.	0.020554	0.998586	0.997172	**k**	0.4258	0.0844	0.0150	Sign.	0.047087	0.992623	0.985245
			**a**	1.0029	0.0205	1.87*10^− 5^	Sign.				**a**	1.0043	0.0470	0.0002	Sign.			
			**b**	0.0046	0.0025	0.1655	**InSign.**				**b**	0.0071	0.0053	0.2711	**InSign.**			
			**n**	1.6846	0.1174	0.0007	Sign.				**n**	1.9569	0.3203	0.0088	Sign.			
		**2**	**k**	0.4267	0.0588	0.0003	Sign.	0.036169	0.992327	0.988491	**k**	0.4700	0.1013	0.0035	Sign.	0.058546	0.979615	0.969423
			**a**	1.0093	0.0360	1.35*10^− 7^	Sign.				**a**	1.0124	0.0583	2.34*10^− 6^	Sign.			
			**b**	0.0040	0.0024	0.1515	**InSign.**				**b**	0.0052	0.0037	0.2142	**InSign.**			
			**n**	1.3602	0.1515	0.0001	Sign.				**n**	1.4142	0.2640	0.0017	Sign.			
		**3**	**k**	0.4290	0.0646	9.48*10^− 5^	Sign.	0.041988	0.986075	0.981433	**k**	0.4926	0.1013	0.0009	Sign.	0.061400	0.969486	0.959314
			**a**	1.0150	0.0417	1.59*10^− 9^	Sign.				**a**	1.0171	0.0611	4.56*10^− 8^	Sign.			
			**b**	0.0025	0.0019	0.2232	**InSign.**				**b**	0.0027	0.0027	0.3480	**InSign.**			
			**n**	1.1605	0.1376	1.45*10^− 5^	Sign.				**n**	1.1056	0.1979	0.0003	Sign.			
	**70**	**1**	**k**	0.3357	0.0449	0.0847	**InSign.**	0.027271	0.999038	0.996153	**k**	0.3668	0.0063	0.0109	Sign.	0.003552	0.999984	0.999938
			**a**	0.9978	0.0272	0.0173	Sign.				**a**	1.0001	0.0036	0.0023	Sign.			
			**b**	-0.0026	0.0068	0.7695	**InSign.**				**b**	0.0027	0.0007	0.1702	**InSign.**			
			**n**	2.0137	0.2025	0.0638	**InSign.**				**n**	2.4458	0.0302	0.0079	Sign.			
		**2**	**k**	0.3459	0.0826	0.0138	Sign.	0.050807	0.989567	0.981742	**k**	0.3267	0.1091	0.0402	Sign.	0.063980	0.983510	0.971142
			**a**	1.0095	0.0505	3.70*10^− 5^	Sign.				**a**	1.0024	0.0640	9.68*10^− 5^	Sign.			
			**b**	0.0069	0.0046	0.2055	**InSign.**				**b**	0.0089	0.0055	0.1824	**InSign.**			
			**n**	1.9265	0.3266	0.0041	Sign.				**n**	2.5862	0.5628	0.0101	Sign.			
		**3**	**k**	0.2801	0.0764	0.0105	Sign.	0.051330	0.986007	0.979010	**k**	0.2629	0.1005	0.0399	Sign.	0.062237	0.979145	0.968717
			**a**	1.0150	0.0507	1.01*10^− 6^	Sign.				**a**	1.0067	0.0621	3.51*10^− 6^	Sign.			
			**b**	0.0064	0.0031	0.0868	**InSign.**				**b**	0.0074	0.0037	0.0932	**InSign.**			
			**n**	2.0167	0.3409	0.0010	Sign.				**n**	2.6188	0.5800	0.0040	Sign.			
**Modified Midilli I**	**50**	**1**	**k**	0.4227	0.0238	2.03*10^− 6^	Sign.	0.017341	0.998150	0.997533	**k**	0.4690	0.0583	0.0002	Sign.	0.038824	0.990610	0.987480
			**b**	0.0038	0.0014	0.0338	Sign.				**b**	0.0053	0.0029	0.1246	**InSign.**			
			**n**	1.4130	0.0729	1.22*10^− 6^	Sign.				**n**	1.5054	0.1832	0.0002	Sign.			
		**2**	**k**	0.4410	0.0398	1.51*10^− 6^	Sign.	0.029429	0.992718	0.991100	**k**	0.4962	0.0688	5.02*10^− 5^	Sign.	0.046313	0.981447	0.977324
			**b**	0.0035	0.0014	0.0393	Sign.				**b**	0.0043	0.0022	0.0800	**InSign.**			
			**n**	1.2554	0.1028	6.63*10^− 7^	Sign.				**n**	1.2705	0.1736	4.48*10^− 5^	Sign.			
		**3**	**k**	0.4748	0.0503	3.54*10^− 7^	Sign.	0.038471	0.982877	0.980243	**k**	0.5355	0.0766	9.45*10^− 6^	Sign.	0.053400	0.966324	0.961143
			**b**	0.0033	0.0013	0.0216	Sign.				**b**	0.0034	0.0017	0.0673	**InSign.**			
			**n**	1.0043	0.0993	1.58*10^− 7^	Sign.				**n**	0.9710	0.1404	1.06*10^− 5^	Sign.			
	**60**	**1**	**k**	0.4019	0.0251	8.93*10^− 5^	Sign.	0.017862	0.998576	0.997864	**k**	0.4215	0.0613	0.0023	Sign.	0.040837	0.992602	0.988902
			**b**	0.0046	0.0022	0.1023	**InSign.**				**b**	0.0071	0.0046	0.1944	**InSign.**			
			**n**	1.6908	0.0955	5.98*10^− 5^	Sign.				**n**	1.9685	0.2626	0.0017	Sign.			
		**2**	**k**	0.4186	0.0454	3.63*10^− 5^	Sign.	0.033676	0.992240	0.990023	**k**	0.4591	0.0794	0.0007	Sign.	0.054415	0.979456	0.973586
			**b**	0.0040	0.0022	0.1179	**InSign.**				**b**	0.0052	0.0035	0.1759	**InSign.**			
			**n**	1.3738	0.1334	1.76*10^− 5^	Sign.				**n**	1.4323	0.2357	0.0005	Sign.			
		**3**	**k**	0.4168	0.0506	9.05*10^− 6^	Sign.	0.040131	0.985866	0.983039	**k**	0.4789	0.0809	0.0001	Sign.	0.058514	0.969208	0.963049
			**b**	0.0025	0.0018	0.1949	**InSign.**				**b**	0.0027	0.0026	0.3190	**InSign.**			
			**n**	1.1769	0.1244	2.65*10^− 6^	Sign.				**n**	1.1206	0.1812	0.0001	Sign.			
	**70**	**1**	**k**	0.3379	0.0260	0.0059	Sign.	0.019348	0.999032	0.998063	**k**	0.3667	0.0037	0.0001	Sign.	0.002512	0.999984	0.999969
			**b**	-0.0026	0.0048	0.6424	**InSign.**				**b**	0.0027	0.0005	0.0355	Sign.			
			**n**	2.0069	0.1308	0.0042	Sign.				**n**	2.4460	0.0198	6.55*10^− 5^	Sign.			
		**2**	**k**	0.3371	0.0607	0.0026	Sign.	0.045648	0.989473	0.985262	**k**	0.3242	0.0793	0.0094	Sign.	0.057235	0.983504	0.976906
			**b**	0.0070	0.0041	0.1507	**InSign.**				**b**	0.0089	0.0049	0.1313	**InSign.**			
			**n**	1.9541	0.2744	0.0008	Sign.				**n**	2.5965	0.4604	0.0024	Sign.			
		**3**	**k**	0.2668	0.0570	0.0023	Sign.	0.047881	0.985795	0.981736	**k**	0.2557	0.0740	0.0106	Sign.	0.057675	0.979105	0.973135
			**b**	0.0065	0.0029	0.0625	**InSign.**				**b**	0.0075	0.0035	0.0679	**InSign.**			
			**n**	2.0673	0.2963	0.0002	Sign.				**n**	2.6582	0.4836	0.0009	Sign.			
**Modified Midilli I I**	**50**	**1**	**k**	0.4289	0.0272	1.88*10^− 5^	Sign.	0.015759	0.998727	0.997963	**k**	0.4725	0.0698	0.0011	Sign.	0.036541	0.993068	0.988909
			**a**	0.9721	0.0182	4.30*10^− 8^	Sign.				**a**	0.9601	0.0406	2.51*10^− 6^	Sign.			
			**b**	0.0304	0.0084	0.0156	Sign.				**b**	0.0447	0.0169	0.0457	Sign.			
			**n**	1.4662	0.0805	9.20*10^− 6^	Sign.				**n**	1.6812	0.2275	0.0007	Sign.			
		**2**	**k**	0.4442	0.0457	1.04*10^− 5^	Sign.	0.026302	0.994830	0.992891	**k**	0.4825	0.0795	0.0003	Sign.	0.041435	0.986799	0.981849
			**a**	0.9664	0.0287	6.58*10^− 10^	Sign.				**a**	0.9534	0.0444	2.31*10^− 8^	Sign.			
			**b**	0.0396	0.0108	0.0063	Sign.				**b**	0.0536	0.0152	0.0079	Sign.			
			**n**	1.3669	0.1197	3.13*10^− 6^	Sign.				**n**	1.5873	0.2384	0.0002	Sign.			
		**3**	**k**	0.4739	0.0595	3.96*10^− 6^	Sign.	0.034385	0.987373	0.984216	**k**	0.5164	0.0939	0.0001	Sign.	0.049501	0.973289	0.966611
			**a**	0.9565	0.0367	6.15*10^− 12^	Sign.				**a**	0.9508	0.0521	4.09*10^− 10^	Sign.			
			**b**	0.0540	0.0119	0.0007	Sign.				**b**	0.0618	0.0157	0.0020	Sign.			
			**n**	1.1689	0.1274	9.03*10^− 7^	Sign.				**n**	1.2893	0.2184	7.22*10^− 5^	Sign.			
	**60**	**1**	**k**	0.4071	0.0311	0.0010	Sign.	0.017616	0.998961	0.997922	**k**	0.4250	0.0755	0.0111	Sign.	0.039516	0.994804	0.989609
			**a**	0.9745	0.0211	2.25*10^− 5^	Sign.				**a**	0.9573	0.0450	0.0002	Sign.			
			**b**	0.0277	0.0110	0.0872	**InSign.**				**b**	0.0447	0.0210	0.1228	**InSign.**			
			**n**	1.7386	0.1124	0.0006	Sign.				**n**	2.1450	0.3195	0.0067	Sign.			
		**2**	**k**	0.4245	0.0559	0.0003	Sign.	0.032325	0.993872	0.990808	**k**	0.4415	0.0977	0.0040	Sign.	0.051410	0.984282	0.976423
			**a**	0.9686	0.0362	1.79*10^− 7^	Sign.				**a**	0.9501	0.0559	2.64*10^− 6^	Sign.			
			**b**	0.0387	0.0152	0.0442	Sign.				**b**	0.0568	0.0210	0.0355	Sign.			
			**n**	1.4742	0.1632	0.0001	Sign.				**n**	1.8276	0.3477	0.0019	Sign.			
		**3**	**k**	0.4251	0.0640	9.48*10^− 5^	Sign.	0.039437	0.987716	0.983621	**k**	0.4801	0.1038	0.0012	Sign.	0.058395	0.972400	0.963200
			**a**	0.9789	0.0432	3.02*10^− 9^	Sign.				**a**	0.9721	0.0631	8.92*10^− 8^	Sign.			
			**b**	0.0344	0.0164	0.0651	**InSign.**				**b**	0.0433	0.0225	0.0869	**InSign.**			
			**n**	1.2595	0.1561	2.07*10^− 5^	Sign.				**n**	1.2981	0.2516	0.0006	Sign.			
	**70**	**1**	**k**	0.3351	0.0432	0.0817	**InSign.**	0.026634	0.999083	0.996330	**k**	0.3672	0.0035	0.0061	Sign.	0.001951	0.999995	0.999981
			**a**	1.0098	0.0396	0.0250	Sign.				**a**	0.9900	0.0025	0.0016	Sign.			
			**b**	-0.0119	0.0275	0.7405	**InSign.**				**b**	0.0100	0.0015	0.0924	**InSign.**			
			**n**	1.9994	0.2070	0.0657	**InSign.**				**n**	2.4631	0.0175	0.0045	Sign.			
		**2**	**k**	0.3335	0.0740	0.0108	Sign.	0.042098	0.992837	0.987465	**k**	0.3299	0.0882	0.0201	Sign.	0.048150	0.990660	0.983655
			**a**	0.9538	0.0472	3.54*10^− 5^	Sign.				**a**	0.9394	0.0528	5.88*10^− 5^	Sign.			
			**b**	0.0514	0.0204	0.0651	**InSign.**				**b**	0.0610	0.0216	0.0479	Sign.			
			**n**	2.1728	0.3396	0.0031	Sign.				**n**	2.8442	0.5060	0.0049	Sign.			
		**3**	**k**	0.2565	0.0642	0.0071	Sign.	0.039187	0.991845	0.987767	**k**	0.2517	0.0764	0.0165	Sign.	0.043930	0.989609	0.984414
			**a**	0.9503	0.0424	5.20*10^− 7^	Sign.				**a**	0.9372	0.0471	1.04*10^− 6^	Sign.			
			**b**	0.0577	0.0157	0.0104	Sign.				**b**	0.0640	0.0167	0.0087	Sign.			
			**n**	2.3526	0.3529	0.0006	Sign.				**n**	2.9576	0.4866	0.0009	Sign.			
**Modified Page**	**50**	**1**	**k**	0.5299	0.0164	7.06*10^− 9^	Sign.	0.023454	0.996052	0.995488	**k**	0.5836	0.0348	6.54*10^− 7^	Sign.	0.043445	0.986282	0.984323
			**n**	1.3207	0.0838	1.01*10^− 6^	Sign.				**n**	1.3450	0.1699	9.73*10^− 5^	Sign.			
		**2**	**k**	0.5053	0.0250	1.97*10^− 9^	Sign.	0.034878	0.988635	0.987498	**k**	0.5540	0.0439	1.80*10^− 7^	Sign.	0.051109	0.974895	0.972384
			**n**	1.1323	0.1002	5.11*10^− 7^	Sign.				**n**	1.0771	0.1473	2.56*10^− 5^	Sign.			
		**3**	**k**	0.4597	0.0334	1.60*10^− 9^	Sign.	0.043783	0.976116	0.974410	**k**	0.5094	0.0537	1.80*10^− 7^	Sign.	0.056855	0.958889	0.955952
			**n**	0.8495	0.0818	5.81*10^− 8^	Sign.				**n**	0.7993	0.1048	2.39*10^− 6^	Sign.			
	**60**	**1**	**k**	0.5699	0.0159	3.19*10^− 7^	Sign.	0.022760	0.997110	0.996532	**k**	0.6228	0.0343	9.26*10^− 6^	Sign.	0.045449	0.988545	0.986254
			**n**	1.6056	0.1071	2.39*10^− 5^	Sign.				**n**	1.7928	0.2555	0.0009	Sign.			
		**2**	**k**	0.5139	0.0256	3.96*10^− 8^	Sign.	0.037163	0.989200	0.987850	**k**	0.5556	0.0449	1.69*10^− 6^	Sign.	0.057200	0.974055	0.970812
			**n**	1.2555	0.1225	7.05*10^− 6^	Sign.				**n**	1.2345	0.1950	0.0002	Sign.			
		**3**	**k**	0.4632	0.0266	2.39*10^− 9^	Sign.	0.041321	0.983517	0.982019	**k**	0.5039	0.0449	2.33*10^− 7^	Sign.	0.058231	0.966456	0.963406
			**n**	1.0888	0.1067	6.01*10^− 7^	Sign.				**n**	1.0151	0.1456	2.35*10^− 5^	Sign.			
	**70**	**1**	**k**	0.5877	0.0111	1.47*10^− 5^	Sign.	0.016984	0.998881	0.998508	**k**	0.6587	0.0056	1.37*10^− 6^	Sign.	0.007742	0.999779	0.999705
			**n**	2.0327	0.1061	0.0003	Sign.				**n**	2.4236	0.0591	3.18*10^− 5^	Sign.			
		**2**	**k**	0.5500	0.0325	2.73*10^− 6^	Sign.	0.051240	0.984082	0.981429	**k**	0.6287	0.0448	8.13*10^− 6^	Sign.	0.066857	0.972990	0.968488
			**n**	1.7494	0.2608	0.0005	Sign.				**n**	2.4325	0.5073	0.0030	Sign.			
		**3**	**k**	0.5057	0.0315	2.25*10^− 7^	Sign.	0.057587	0.976517	0.973581	**k**	0.5803	0.0407	5.76*10^− 7^	Sign.	0.069326	0.965498	0.961185
			**n**	1.8408	0.2992	0.0003	Sign.				**n**	2.4318	0.5322	0.0018	Sign.			
**Page**	**50**	**1**	**k**	0.4323	0.0318	2.72*10^− 6^	Sign.	0.023454	0.996052	0.995488	**k**	0.4847	0.0646	0.0001	Sign.	0.043445	0.986282	0.984323
			**n**	1.3207	0.0838	1.01*10^− 6^	Sign.				**n**	1.3450	0.1699	9.73*10^− 5^	Sign.			
		**2**	**k**	0.4617	0.0466	1.72*10^− 6^	Sign.	0.034878	0.988635	0.987498	**k**	0.5294	0.0751	3.50*10^− 5^	Sign.	0.051109	0.974895	0.972384
			**n**	1.1323	0.1002	5.11*10^− 7^	Sign.				**n**	1.0771	0.1473	2.56*10^− 5^	Sign.			
		**3**	**k**	0.5167	0.0560	2.49*10^− 7^	Sign.	0.043783	0.976116	0.974410	**k**	0.5832	0.0796	3.75*10^− 6^	Sign.	0.056855	0.958889	0.955952
			**n**	0.8495	0.0818	5.81*10^− 8^	Sign.				**n**	0.7993	0.1048	2.39*10^− 6^	Sign.			
	**60**	**1**	**k**	0.4054	0.0317	5.17*10^− 5^	Sign.	0.022760	0.997110	0.996532	**k**	0.4278	0.0674	0.0014	Sign.	0.045449	0.988545	0.986254
			**n**	1.6056	0.1071	2.39*10^− 5^	Sign.				**n**	1.7928	0.2555	0.0009	Sign.			
		**2**	**k**	0.4336	0.0494	2.22*10^− 5^	Sign.	0.037163	0.989200	0.987850	**k**	0.4840	0.0823	0.0004	Sign.	0.057200	0.974055	0.970812
			**n**	1.2555	0.1225	7.05*10^− 6^	Sign.				**n**	1.2345	0.1950	0.0002	Sign.			
		**3**	**k**	0.4326	0.0512	3.86*10^− 6^	Sign.	0.041321	0.983517	0.982019	**k**	0.4987	0.0790	5.73*10^− 5^	Sign.	0.058231	0.966456	0.963406
			**n**	1.0888	0.1067	6.01*10^− 7^	Sign.				**n**	1.0151	0.1456	2.35*10^− 5^	Sign.			
	**70**	**1**	**k**	0.3394	0.0229	0.0007	Sign.	0.016984	0.998881	0.998508	**k**	0.3636	0.0111	6.28*10^− 5^	Sign.	0.007742	0.999779	0.999705
			**n**	2.0327	0.1061	0.0003	Sign.				**n**	2.4236	0.0591	3.18*10^− 5^	Sign.			
		**2**	**k**	0.3513	0.0671	0.0019	Sign.	0.051240	0.984082	0.981429	**k**	0.3234	0.0919	0.0125	Sign.	0.066857	0.972990	0.968488
			**n**	1.7494	0.2608	0.0005	Sign.				**n**	2.4325	0.5073	0.0030	Sign.			
		**3**	**k**	0.2851	0.0676	0.0029	Sign.	0.057587	0.976517	0.973581	**k**	0.2662	0.0884	0.0167	Sign.	0.069326	0.965498	0.961185
			**n**	1.8408	0.2992	0.0003	Sign.				**n**	2.4318	0.5322	0.0018	Sign.			
**Wang-Sigh**	**50**	**1**	**b**	-0.3509	0.0195	4.05*10^− 7^	Sign.	0.069024	0.965806	0.960921	**b**	-0.3629	0.0267	2.75*10^− 6^	Sign.	0.094503	0.935093	0.925820
			**a**	0.0296	0.0030	2.19*10^− 5^	Sign.				**a**	0.0314	0.0041	0.0001	Sign.			
		**2**	**b**	-0.2788	0.0200	7.04*10^− 8^	Sign.	0.111925	0.882963	0.871260	**b**	-0.2836	0.0237	2.98*10^− 7^	Sign.	0.132561	0.831113	0.814224
			**a**	0.0182	0.0022	1.06*10^− 5^	Sign.				**a**	0.0188	0.0027	3.54E*10^− 5^	Sign.			
		**3**	**b**	-0.2108	0.0165	4.31*10^− 9^	Sign.	0.145251	0.737131	0.718355	**b**	-0.2139	0.0184	1.42*10^− 8^	Sign.	0.161656	0.667642	0.643902
			**a**	0.0104	0.0014	2.71*10^− 6^	Sign.				**a**	0.0106	0.0015	6.98*10^− 6^	Sign.			
	**60**	**1**	**b**	-0.4198	0.0222	7.66*10^− 6^	Sign.	0.052189	0.984803	0.981764	**b**	-0.4387	0.0334	4.58*10^− 5^	Sign.	0.078475	0.965848	0.959018
			**a**	0.0434	0.0044	0.0002	Sign.				**a**	0.0469	0.0067	0.0009	Sign.			
		**2**	**b**	-0.3208	0.0212	3.64*10^− 7^	Sign.	0.088992	0.938070	0.930329	**b**	-0.3284	0.0264	1.62*10^− 6^	Sign.	0.110552	0.903087	0.890972
			**a**	0.0246	0.0029	2.84*10^− 5^	Sign.				**a**	0.0256	0.0036	0.0001	Sign.			
		**3**	**b**	-0.2544	0.0179	1.97*10^− 8^	Sign.	0.113403	0.875854	0.864568	**b**	-0.2578	0.0210	9.33*10^− 8^	Sign.	0.133469	0.823770	0.807749
			**a**	0.0152	0.0018	5.13*10^− 6^	Sign.				**a**	0.0155	0.0022	1.93*10^− 5^	Sign.			
	**70**	**1**	**b**	-0.4480	0.0579	0.0045	Sign.	0.076568	0.977254	0.969672	**b**	-0.5146	0.0764	0.0067	Sign.	0.101162	0.962186	0.949582
			**a**	0.0475	0.0168	0.0669	**InSign.**				**a**	0.0650	0.0223	0.0615	**InSign.**			
		**2**	**b**	-0.3799	0.0292	1.27*10^− 5^	Sign.	0.085337	0.955849	0.948490	**b**	-0.3948	0.0381	4.70*10^− 5^	Sign.	0.111296	0.925150	0.912676
			**a**	0.0352	0.0050	0.0004	Sign.				**a**	0.0377	0.0066	0.0012	Sign.			
		**3**	**b**	-0.3181	0.0248	1.28*10^− 6^	Sign.	0.103870	0.923602	0.914052	**b**	-0.3272	0.0297	4.08*10^− 6^	Sign.	0.124377	0.888945	0.875063
			**a**	0.0243	0.0034	9.34*10^− 5^	Sign.				**a**	0.0255	0.0040	0.0002	Sign.			
**Weibullian**	**50**	**1**	**β**	1.3207	0.0838	1.01*10^− 6^	Sign.	0.023454	0.996052	0.995488	**β**	1.3450	0.1699	9.73*10^− 5^	Sign.	0.043445	0.986282	0.984323
			**a**	1.8871	0.0584	7.06*10^− 9^	Sign.				**a**	1.7134	0.1021	6.54*10^− 7^	Sign.			
		**2**	**β**	1.1323	0.1002	5.11*10^− 7^	Sign.	0.034878	0.988635	0.987498	**β**	1.0771	0.1473	2.56*10^− 5^	Sign.	0.051109	0.974895	0.972384
			**a**	1.9788	0.0981	1.97*10^− 9^	Sign.				**a**	1.8049	0.1429	1.80*10^− 7^	Sign.			
		**3**	**β**	0.8495	0.0818	5.81*10^− 8^	Sign.	0.043783	0.976116	0.974410	**β**	0.7993	0.1048	2.39*10^− 6^	Sign.	0.056855	0.958889	0.955952
			**a**	2.1754	0.1582	1.60*10^− 9^	Sign.				**a**	1.9632	0.2071	1.80*10^− 7^	Sign.			
	**60**	**1**	**β**	1.6056	0.1071	2.39*10^− 5^	Sign.	0.022760	0.997110	0.996532	**β**	1.7928	0.2555	0.0009	Sign.	0.045449	0.988545	0.986254
			**a**	1.7548	0.0490	3.19*10^− 7^	Sign.				**a**	1.6058	0.0883	9.26*10^− 6^	Sign.			
		**2**	**β**	1.2555	0.1225	7.05*10^− 6^	Sign.	0.037163	0.989200	0.987850	**β**	1.2345	0.1950	0.0002	Sign.	0.057200	0.974055	0.970812
			**a**	1.9458	0.0969	3.96*10^− 8^	Sign.				**a**	1.8000	0.1453	1.69*10^− 6^	Sign.			
		**3**	**β**	1.0888	0.1067	6.01*10^− 7^	Sign.	0.041321	0.983517	0.982019	**β**	1.0151	0.1456	2.35*10^− 5^	Sign.	0.058231	0.966456	0.963406
			**a**	2.1589	0.1242	2.39*10^− 9^	Sign.				**a**	1.9844	0.1770	2.33*10^− 7^	Sign.			
	**70**	**1**	**β**	2.0327	0.1061	0.0003	Sign.	0.016984	0.998881	0.998508	**β**	2.4236	0.0591	3.18*10^− 5^	Sign.	0.007742	0.999779	0.999705
			**a**	1.7016	0.0321	1.47*10^− 5^	Sign.				**a**	1.5181	0.0129	1.37*10^− 6^	Sign.			
		**2**	**β**	1.7494	0.2608	0.0005	Sign.	0.051240	0.984082	0.981429	**β**	2.4325	0.5073	0.0030	Sign.	0.066857	0.972990	0.968488
			**a**	1.8183	0.1075	2.73*10^− 6^	Sign.				**a**	1.5906	0.1133	8.13*10^− 6^	Sign.			
		**3**	**β**	1.8408	0.2992	0.0003	Sign.	0.057587	0.976517	0.973581	**β**	2.4318	0.5322	0.0018	Sign.	0.069326	0.965498	0.961185
			**a**	1.9775	0.1230	2.25*10^− 7^	Sign.				**a**	1.7233	0.1210	5.76*10^− 7^	Sign.			
**Weibullian I**	**50**	**1**	**n**	1.3207	0.0838	1.01*10^− 6^	Sign.	0.023454	0.996052	0.995488	**n**	1.3450	0.1699	9.73*10^− 5^	Sign.	0.043445	0.986282	0.984323
			**δ**	3.5485	0.1610	9.99*10^− 8^	Sign.				**δ**	3.1854	0.2815	9.42*10^− 6^	Sign.			
		**2**	**n**	1.1323	0.1002	5.11*10^− 7^	Sign.	0.034878	0.988635	0.987498	**n**	1.0771	0.1473	2.56*10^− 5^	Sign.	0.051109	0.974895	0.972384
			**δ**	4.1335	0.2840	4.67*10^− 8^	Sign.				**δ**	3.9151	0.4200	3.02*10^− 6^	Sign.			
		**3**	**n**	0.8495	0.0818	5.81*10^− 8^	Sign.	0.043783	0.976116	0.974410	**n**	0.7993	0.1048	2.39*10^− 6^	Sign.	0.056855	0.958889	0.955952
			**δ**	5.8069	0.5006	1.44*10^− 8^	Sign.				**δ**	5.5736	0.6611	7.40*10^− 7^	Sign.			
	**60**	**1**	**n**	1.6056	0.1071	2.39*10^− 5^	Sign.	0.022760	0.997110	0.996532	**n**	1.7928	0.2555	0.0009	Sign.	0.045449	0.988545	0.986254
			**δ**	2.9500	0.1257	2.61*10^− 6^	Sign.				**δ**	2.5569	0.2181	7.94*10^− 5^	Sign.			
		**2**	**n**	1.2555	0.1225	7.05*10^− 6^	Sign.	0.037163	0.989200	0.987850	**n**	1.2345	0.1950	0.0002	Sign.	0.057200	0.974055	0.970812
			**δ**	3.7810	0.2715	6.84*10^− 7^	Sign.				**δ**	3.5373	0.4109	2.56*10^− 5^	Sign.			
		**3**	**n**	1.0888	0.1067	6.01*10^− 7^	Sign.	0.041321	0.983517	0.982019	**n**	1.0151	0.1456	2.35*10^− 5^	Sign.	0.058231	0.966456	0.963406
			**δ**	4.6440	0.3637	6.13*10^− 8^	Sign.				**δ**	4.5130	0.5295	3.56*10^− 6^	Sign.			
	**70**	**1**	**n**	2.0327	0.1061	0.0003	Sign.	0.016984	0.998881	0.998508	**n**	2.4236	0.0591	3.18*10^− 5^	Sign.	0.007742	0.999779	0.999705
			**δ**	2.5649	0.0738	5.24*10^− 5^	Sign.				**δ**	2.1416	0.0269	4.35*10^− 6^	Sign.			
		**2**	**n**	1.7494	0.2608	0.0005	Sign.	0.051240	0.984082	0.981429	**n**	2.4325	0.5073	0.0030	Sign.	0.066857	0.972990	0.968488
			**δ**	2.9290	0.2648	3.25*10^− 5^	Sign.				**δ**	2.2412	0.2326	7.16*10^− 5^	Sign.			
		**3**	**n**	1.8408	0.2992	0.0003	Sign.	0.057587	0.976517	0.973581	**n**	2.4318	0.5322	0.0018	Sign.	0.069326	0.965498	0.961185
			**δ**	3.1109	0.2953	5.75*10^− 6^	Sign.				**δ**	2.4283	0.2532	1.16*10^− 5^	Sign.			

MMs: mathematical models. DAT: drying air temperature, ºC; LT; layer thickness, cm: k_1_, k_2_ and k: drying constants, h^− 1^; a, b, c, n, ɤ, β and δ: mathematical models constants or parameters, dimensionless; S.E: Standard error; Sign. – InSign: Significant – InSignificant at *p* ≤ 0.05; RMSE: The root mean square error; R^2^: The coefficient of determination; R^2^_adj_.: The adjusted coefficient of determination.

### Thermodynamic properties

Table 5 delineates the thermodynamic parameters (enthalpy, entropy, and Gibbs free energy) of the OD and HSD for drying of pomegranate peels at differing drying temperatures and layer thicknesses.

**Table 5:** Thermodynamic properties of pomegranate peels for OD and HSD under different drying air temperatures and different layers thicknesses.

Drying system	DAT, °C	LT, cm	ΔH, kJ mol^− 1^	ΔS, kJ mol^− 1^ K^− 1^	ΔG, kJ mol^− 1^
Oven dryer (OD)	50	1	40.863	-0.2113	109.126
		2	23.075	-0.1653	76.465
		3	26.373	-0.1792	84.247
	60	1	40.780	-0.2116	111.240
		2	22.992	-0.1655	78.119
		3	26.290	-0.1794	86.040
	70	1	40.697	-0.2118	113.357
		2	22.909	-0.1658	79.776
		3	26.206	-0.1797	87.835
Hybrid solar dryer (HSD)	50	1	38.401	-0.2036	104.179
		2	23.131	-0.1649	76.381
		3	26.034	-0.1780	83.520
	60	1	38.317	-0.2039	106.217
		2	23.048	-0.1651	78.030
		3	25.951	-0.1782	85.301
	70	1	38.234	-0.2041	108.257
		2	22.965	-0.1654	79.683
		3	25.868	-0.1785	87.085

The data presented in the table show the enthalpy, entropy, and Gibbs free energy values at various drying temperatures. As the drying temperature rises, both enthalpy and entropy decrease, whereas Gibbs free energy increases. Enthalpy reflects the energy needed to remove water bound to the dry matter during drying, and it tends to decrease with higher drying temperatures^99,100^. Lower enthalpy at reduced temperatures signifies greater energy demand for drying tamarind seeds, a trend also seen in studies on Baru fruits by Resende et al.^67^Bode pepper grains by Rodovalho et al.^22^and tamarind seeds by Ferreira Junior et al.^101^. Entropy values (Table 5) decline as drying temperature increases because higher temperatures greatly stimulate the product’s water molecules, reducing the overall order in the water-product system^101,102^. Entropy, which measures the disorder between water and the product^101,103^shows lower values at elevated temperatures. Negative entropy values are often linked to chemical adsorption or structural changes in the adsorbent material^104^. Meanwhile, positive Gibbs free energy values (Table 5) point to an endergonic process, where energy input into the drying air is required for moisture removal. Similar patterns have been reported in other studies^100,105,106^.

## Conclusion

In current study, pomegranate peels were dried using a HSD at three different temperatures of 50, 60, and 70 °C and three different layer thicknesses of 1, 2, and 3 cm. Then, the OD was used to compare the performance of the drying process with the HSD in terms of drying characteristics, EMD, mathematical modeling, activation energy, and thermodynamic properties. The obtained results showed that the drying process using OD led to a decrease in the MC of all pomegranate peel samples from 325.53% to average MC of 2.82, 2.28, and 2.18%, at layer thicknesses of 1, 2, and 3 cm, respectively. Furthermore, drying of pomegranate peel using HSD led to decrease the MC up to 3.08, 3.16, and 2.33%, at layer thicknesses of 1, 2, and 3 cm, respectively. On the other hand, raising the air temperature from 50 to 70 °C leads to a reduction in the time required for the finished product to dry by about 100%, 57%, and 67%, for layer thicknesses of 1 cm, 2 cm, and 3 cm, respectively, for both drying methods. Additionally, the maximum EMD during the current study 12.17 × 10^− 9^ m² s⁻¹, and it was observed at a drying temperature of 70 °C, and 3 cm layer thickness. Furthermore, the activation energy values for thicknesses of 1.0, 2.0, and 3.0 cm were 43.55, 25.76, and 29.06 kJ mol⁻¹, respectively, for the OD, and 41.09, 25.82, and 28.72 kJ mol⁻¹, respectively, for the HSD. In addition, Modified Midilli (II) was the best mathematical model to show how pomegranate peels dried by both drying systems. Moreover, results showed that with the increase in drying temperature, enthalpy and entropy decreased, while Gibbs free energy increased.

### Practical applications

The results demonstrate that optimized drying conditions significantly enhance the preservation, structural integrity, and overall quality of pomegranate peels, making them more suitable for use as functional ingredients in the agri-food, nutraceutical, and cosmetic industries. By maintaining key physicochemical and thermodynamic characteristics during drying, these by-products can be effectively repurposed into value-added products, contributing to waste reduction and sustainable processing.

### Future work

Future research should prioritize the evaluation of bioactive compound retention, antioxidant stability, and the incorporation of dried peels into novel product formulations. Additionally, efforts should be directed toward scaling up hybrid drying technologies for cost-effective and energy-efficient industrial applications.

## Electronic supplementary material

Below is the link to the electronic supplementary material.

Supplementary Material 1

## Data Availability

All data is provided within the article.
