# Supplementary material for: Comparative analysis of drying kinetics, thermodynamic properties, and mathematical modeling of pomegranate peel (Punica granatum L.) in a hybrid solar dryer and an oven dryer
Source: Sci Rep. 2025 Jul 19;15:26288. doi: 10.1038/s41598-025-11016-8 (PMC12276303; doi:10.1038/s41598-025-11016-8)
Supplement: Supplementary file 1 — Supplementary Material 1 [file 41598_2025_11016_MOESM1_ESM.docx]

**Table S1.** Moisture content (d.b.) of pomegranate peels at different drying temperatures and layers thicknesses for oven dryer (OD).

| Drying time, h | DAT50 | | | DAT60 | | | DAT70 | | |
| --- | --- | --- | --- | --- | --- | --- | --- | --- | --- |
|  | **LT1** | **LT2** | **LT3** | **LT1** | **LT2** | **LT3** | **LT1** | **LT2** | **LT3** |
| 0 | 325.53 | 325.5319 | 325.5319 | 325.5319 | 325.5319 | 325.5319 | 325.5319 | 325.5319 | 325.5319 |
| 1 | 218.883 | 221.9251 | 226.9444 | 222.5201 | 227.6139 | 237.5367 | 229.3296 | 243.3628 | 263.754 |
| 2 | 102.778 | 104.0642 | 106.0638 | 86.01173 | 95.95174 | 102.9223 | 85.95469 | 77.78761 | 93.96648 |
| 3 | 45.2778 | 50.80214 | 66.75532 | 33.43109 | 51.20643 | 65.95174 | 5.825243 | 41.29794 | 44.13408 |
| 4 | 27.7778 | 39.03743 | 55.58511 | 17.0088 | 38.06971 | 51.47453 | 1.618123 | 27.72861 | 32.68156 |
| 5 | 18.3333 | 28.60963 | 38.56383 | 9.384164 | 23.59249 | 34.31635 |  | 17.9941 | 25.97765 |
| 6 | 11.3889 | 22.19251 | 33.7766 | 3.225806 | 15.81769 | 24.93298 |  | 8.849558 | 20.39106 |
| 7 | 8.05556 | 16.04278 | 29.25532 |  | 10.99196 | 20.37534 |  | 1.769912 | 14.24581 |
| 8 | 3.61111 | 12.29947 | 25 |  | 6.434316 | 13.13673 |  |  | 6.98324 |
| 9 |  | 9.625668 | 24.46809 |  | 2.412869 | 9.383378 |  |  | 3.072626 |
| 10 |  | 5.614973 | 21.54255 |  |  | 5.898123 |  |  |  |
| 11 |  | 2.673797 | 19.14894 |  |  | 3.217158 |  |  |  |
| 12 |  |  | 14.09574 |  |  | 1.340483 |  |  |  |
| 13 |  |  | 9.840426 |  |  |  |  |  |  |
| 14 |  |  | 5.851064 |  |  |  |  |  |  |
| 15 |  |  | 2.12766 |  |  |  |  |  |  |

**Table S2.** Moisture content (d.b.) of pomegranate peels at different drying temperatures and layers thicknesses for hybrid solar dryer (HSD).

| Drying time, h | DAT50 | | | DAT60 | | | DAT70 | | | |
| --- | --- | --- | --- | --- | --- | --- | --- | --- | --- | --- |
|  | **LT1** | **LT2** | **LT3** | **LT1** | **LT2** | **LT3** | **LT1** | **LT2** | **LT3** |  |
| 0 | 325.532 | 325.5319 | 325.5319 | 325.5319 | 325.5319 | 325.5319 | 325.5319 | 325.5319 | 325.5319 |  |
| 1 | 215.104 | 217.6781 | 224.2507 | 220.1592 | 224.761 | 232.7635 | 226.5193 | 239.9417 | 258.6751 |  |
| 2 | 70.5722 | 72.29551 | 73.17708 | 57.26496 | 66.66667 | 72.67905 | 45.74132 | 47.81341 | 62.70718 |  |
| 3 | 44.1417 | 50.65963 | 65.625 | 35.32764 | 53.06667 | 66.04775 | 5.047319 | 42.27405 | 43.92265 |  |
| 4 | 28.3379 | 39.31398 | 54.42708 | 17.94872 | 39.73333 | 51.45889 | 2.839117 | 27.98834 | 32.59669 |  |
| 5 | 18.5286 | 29.02375 | 37.76042 | 9.116809 | 22.93333 | 35.01326 |  | 18.0758 | 26.24309 |  |
| 6 | 11.4441 | 23.7467 | 33.59375 | 3.133903 | 15.2 | 25.46419 |  | 9.329446 | 20.71823 |  |
| 7 | 8.44687 | 16.62269 | 29.16667 |  | 11.73333 | 21.48541 |  | 2.332362 | 12.98343 |  |
| 8 | 3.26976 | 13.45646 | 24.21875 |  | 7.2 | 13.52785 |  |  | 7.18232 |  |
| 9 |  | 10.55409 | 22.91667 |  | 3.733333 | 10.07958 |  |  | 3.59116 |  |
| 10 |  | 6.068602 | 20.57292 |  |  | 4.774536 |  |  |  |  |
| 11 |  | 3.430079 | 17.96875 |  |  | 2.917772 |  |  |  |  |
| 12 |  |  | 14.32292 |  |  | 1.061008 |  |  |  |  |
| 13 |  |  | 10.15625 |  |  |  |  |  |  |  |
| 14 |  |  | 5.989583 |  |  |  |  |  |  |  |
| 15 |  |  | 2.34375 |  |  |  |  |  |  |  |

**Table S3.** Moisture ratio of pomegranate peels at different drying temperatures and layers thicknesses for oven dryer (OD).

| Drying time, h | DAT50 | | | DAT60 | | | DAT70 | | |
| --- | --- | --- | --- | --- | --- | --- | --- | --- | --- |
|  | **LT1** | **LT2** | **LT3** | **LT1** | **LT2** | **LT3** | **LT1** | **LT2** | **LT3** |
| 0 | 1.00 | 1 | 1 | 1 | 1 | 1 | 1 | 1 | 1 |
| 1 | 0.672386 | 0.681731 | 0.697149 | 0.683558 | 0.699206 | 0.729688 | 0.704477 | 0.747585 | 0.810225 |
| 2 | 0.315723 | 0.319674 | 0.325817 | 0.264219 | 0.294754 | 0.316167 | 0.264044 | 0.238955 | 0.288655 |
| 3 | 0.139089 | 0.156059 | 0.205065 | 0.102697 | 0.157301 | 0.202597 | 0.017895 | 0.126863 | 0.135575 |
| 4 | 0.08533 | 0.119919 | 0.170752 | 0.052249 | 0.116946 | 0.158124 | 0.004971 | 0.085179 | 0.100394 |
| 5 | 0.056318 | 0.087886 | 0.118464 | 0.028827 | 0.072474 | 0.105416 |  | 0.055276 | 0.079801 |
| 6 | 0.034985 | 0.068173 | 0.103758 | 0.009909 | 0.04859 | 0.076592 |  | 0.027185 | 0.062639 |
| 7 | 0.024746 | 0.049282 | 0.089869 |  | 0.033766 | 0.062591 |  | 0.005437 | 0.043762 |
| 8 | 0.011093 | 0.037783 | 0.076797 |  | 0.019766 | 0.040355 |  |  | 0.021452 |
| 9 |  | 0.029569 | 0.075163 |  | 0.007412 | 0.028825 |  |  | 0.009439 |
| 10 |  | 0.017249 | 0.066176 |  |  | 0.018118 |  |  |  |
| 11 |  | 0.008214 | 0.058824 |  |  | 0.009883 |  |  |  |
| 12 |  |  | 0.043301 |  |  | 0.004118 |  |  |  |
| 13 |  |  | 0.030229 |  |  |  |  |  |  |
| 14 |  |  | 0.017974 |  |  |  |  |  |  |
| 15 |  |  | 0.006536 |  |  |  |  |  |  |

**Table S4.** Moisture ratio of pomegranate peels at different drying temperatures and layers thicknesses for hybrid solar dryer (HSD).

| Drying time, h | DAT50 | | | DAT60 | | | DAT70 | | |
| --- | --- | --- | --- | --- | --- | --- | --- | --- | --- |
|  | **LT1** | **LT2** | **LT3** | **LT1** | **LT2** | **LT3** | **LT1** | **LT2** | **LT3** |
| 0 | 1.00 | 1 | 1 | 1 | 1 | 1 | 1 | 1 | 1 |
| 1 | 0.660778 | 0.668684 | 0.688875 | 0.676306 | 0.690442 | 0.715025 | 0.695844 | 0.737076 | 0.794623 |
| 2 | 0.21679 | 0.222084 | 0.224792 | 0.175912 | 0.204793 | 0.223262 | 0.140513 | 0.146878 | 0.19263 |
| 3 | 0.135599 | 0.155621 | 0.201593 | 0.108523 | 0.163015 | 0.202892 | 0.015505 | 0.129861 | 0.134926 |
| 4 | 0.087051 | 0.120768 | 0.167194 | 0.055137 | 0.122057 | 0.158076 | 0.008721 | 0.085977 | 0.100134 |
| 5 | 0.056918 | 0.089158 | 0.115996 | 0.028006 | 0.070449 | 0.107557 |  | 0.055527 | 0.080616 |
| 6 | 0.035155 | 0.072947 | 0.103196 | 0.009627 | 0.046693 | 0.078223 |  | 0.028659 | 0.063644 |
| 7 | 0.025948 | 0.051063 | 0.089597 |  | 0.036044 | 0.066001 |  | 0.007165 | 0.039884 |
| 8 | 0.010044 | 0.041337 | 0.074397 |  | 0.022118 | 0.041556 |  |  | 0.022063 |
| 9 |  | 0.032421 | 0.070398 |  | 0.011468 | 0.030963 |  |  | 0.011032 |
| 10 |  | 0.018642 | 0.063198 |  |  | 0.014667 |  |  |  |
| 11 |  | 0.010537 | 0.055198 |  |  | 0.008963 |  |  |  |
| 12 |  |  | 0.043999 |  |  | 0.003259 |  |  |  |
| 13 |  |  | 0.031199 |  |  |  |  |  |  |
| 14 |  |  | 0.018399 |  |  |  |  |  |  |
| 15 |  |  | 0.0072 |  |  |  |  |  |  |

**Table S5.** Drying rate of pomegranate peels at different drying temperatures and layers thicknesses for oven dryer (OD).

| Drying time, h | DAT50 | | | DAT60 | | | DAT70 | | |
| --- | --- | --- | --- | --- | --- | --- | --- | --- | --- |
|  | **LT1** | **LT2** | **LT3** | **LT1** | **LT2** | **LT3** | **LT1** | **LT2** | **LT3** |
| 0 | 0 | 0 | 0 | 0 | 0 | 0 | 0 | 0 | 0 |
| 1 | 106.65 | 103.61 | 98.59 | 103.01 | 97.92 | 88.00 | 96.20 | 82.17 | 61.78 |
| 2 | 116.11 | 117.86 | 120.88 | 136.51 | 131.66 | 134.61 | 143.37 | 165.58 | 169.79 |
| 3 | 57.50 | 53.26 | 39.31 | 52.58 | 44.75 | 36.97 | 80.13 | 36.49 | 49.83 |
| 4 | 17.50 | 11.76 | 11.17 | 16.42 | 13.14 | 14.48 | 4.21 | 13.57 | 11.45 |
| 5 | 9.44 | 10.43 | 17.02 | 7.62 | 14.48 | 17.16 |  | 9.73 | 6.70 |
| 6 | 6.94 | 6.42 | 4.79 | 6.16 | 7.77 | 9.38 |  | 9.14 | 5.59 |
| 7 | 3.33 | 6.15 | 4.52 |  | 4.83 | 4.56 |  | 7.08 | 6.15 |
| 8 | 4.44 | 3.74 | 4.26 |  | 4.56 | 7.24 |  |  | 7.26 |
| 9 |  | 2.67 | 0.53 |  | 4.02 | 3.75 |  |  | 3.91 |
| 10 |  | 4.01 | 2.93 |  |  | 3.49 |  |  |  |
| 11 |  | 2.94 | 2.39 |  |  | 2.68 |  |  |  |
| 12 |  |  | 5.05 |  |  | 1.88 |  |  |  |
| 13 |  |  | 4.26 |  |  |  |  |  |  |
| 14 |  |  | 3.99 |  |  |  |  |  |  |
| 15 |  |  | 3.72 |  |  |  |  |  |  |

**Table S6.** Drying rate of pomegranate peels at different drying temperatures and layers thicknesses for hybrid solar dryer (HSD).

| Drying time, h | DAT50 | | | DAT60 | | | DAT70 | | |
| --- | --- | --- | --- | --- | --- | --- | --- | --- | --- |
|  | **LT1** | **LT2** | **LT3** | **LT1** | **LT2** | **LT3** | **LT1** | **LT2** | **LT3** |
| 0 | 0 | 0 | 0 | 0 | 0 | 0 | 0 | 0 | 0 |
| 1 | 110.4277 | 107.8538 | 101.2812 | 105.3727 | 100.7709 | 92.76841 | 99.01261 | 85.59021 | 66.85681 |
| 2 | 144.532 | 145.3826 | 151.0736 | 162.8942 | 158.0943 | 160.0845 | 180.778 | 192.1283 | 195.9679 |
| 3 | 26.43052 | 21.63588 | 7.55208 | 21.93732 | 13.6 | 6.6313 | 40.694 | 5.53936 | 18.78453 |
| 4 | 15.80382 | 11.34565 | 11.19792 | 17.37892 | 13.33334 | 14.58886 | 2.208202 | 14.28571 | 11.32596 |
| 5 | 9.80926 | 10.29023 | 16.66666 | 8.831911 | 16.8 | 16.44563 |  | 9.91254 | 6.3536 |
| 6 | 7.08447 | 5.27705 | 4.16667 | 5.982906 | 7.73333 | 9.54907 |  | 8.746354 | 5.52486 |
| 7 | 2.997274 | 7.12401 | 4.42708 |  | 3.46667 | 3.97878 |  | 6.997084 | 7.7348 |
| 8 | 5.177111 | 3.16623 | 4.94792 |  | 4.53333 | 7.95756 |  |  | 5.80111 |
| 9 |  | 2.90237 | 1.30208 |  | 3.466667 | 3.44827 |  |  | 3.59116 |
| 10 |  | 4.485488 | 2.34375 |  |  | 5.305044 |  |  |  |
| 11 |  | 2.638523 | 2.60417 |  |  | 1.856764 |  |  |  |
| 12 |  |  | 3.64583 |  |  | 1.856764 |  |  |  |
| 13 |  |  | 4.16667 |  |  |  |  |  |  |
| 14 |  |  | 4.166667 |  |  |  |  |  |  |
| 15 |  |  | 3.645833 |  |  |  |  |  |  |
